# Using RE-AIM to examine the potential public health impact of an integrated collaborative care intervention for weight and depression management in primary care: Results from the RAINBOW trial

**DOI:** 10.1371/journal.pone.0248339

**Published:** 2021-03-11

**Authors:** Megan A. Lewis, Laura K. Wagner, Lisa G. Rosas, Nan Lv, Elizabeth M. Venditti, Lesley E. Steinman, Bryan J. Weiner, Jeremy D. Goldhaber-Fiebert, Mark B. Snowden, Jun Ma

**Affiliations:** 1 Center for Communication Science, RTI International, Seattle, WA, United States of America; 2 Center for Communication Science, RTI International, Research Triangle Park, NC, United States of America; 3 Department of Epidemiology and Population Health, Stanford School of Medicine, Palo Alto, CA, United States of America; 4 Institute of Health Research and Policy, University of Illinois at Chicago, Chicago, IL, United States of America; 5 Department of Psychiatry, University of Pittsburgh School of Medicine, Pittsburgh, PA, United States of America; 6 Health Promotion Research Center, University of Washington, Seattle, WA, United States of America; 7 Department of Global Health, University of Washington, Seattle, WA, United States of America; 8 Department of Health Services, University of Washington, Seattle, WA, United States of America; 9 Stanford Health Policy, Centers for Health Policy and Primary Care and Outcomes Research, Stanford University, Palo Alto, CA, United States of America; 10 Department of Psychiatry and Behavioral Sciences, School of Medicine, University of Washington, Seattle, WA, United States of America; 11 Department of Medicine, College of Medicine, University of Illinois at Chicago, Chicago, IL, United States of America; University of Arkansas for Medical Sciences, UNITED STATES

## Abstract

**Background:**

An integrated collaborative care intervention was used to treat primary care patients with comorbid obesity and depression in a randomized clinical trial. To increase wider uptake and dissemination, information is needed on translational potential.

**Methods:**

The trial collected longitudinal, qualitative data at baseline, 6 months (end of intensive treatment), 12 months (end of maintenance treatment), and 24 months (end of follow-up). Semi-structured interviews (n = 142) were conducted with 54 out of 409 randomly selected trial participants and 37 other stakeholders, such as recruitment staff, intervention staff, and clinicians. Using a Framework Analysis approach, we examined themes across time and stakeholder groups according to the RE-AIM (Reach, Effectiveness, Adoption, Implementation, and Maintenance) framework.

**Results:**

At baseline, participants and other stakeholders reported being skeptical of the collaborative care approach related to some RE-AIM dimensions. However, over time they indicated greater confidence regarding the potential for future public health impact. They also provided information on barriers and actionable information to enhance program reach, effectiveness, adoption, implementation, and maintenance.

**Conclusions:**

RE-AIM provided a useful framework for understanding how to increase the impact of a collaborative and integrative approach for treating comorbid obesity and depression. It also demonstrates the utility of using the framework as a planning tool early in the evidence-generation pipeline.

## Introduction

Obesity and depression are highly prevalent conditions [1] that commonly present together among patients in primary care, although typically they are treated separately [2]. Adults with obesity are 32% more likely to experience depression compared with adults of normal weight, [3] and adults with depression are 58% more likely to be obese as compared with adults without depression [4]. The potential public health impact of these comorbid conditions is great given their association with prevalent chronic illnesses such as type 2 diabetes and cardiovascular disease, which are leading causes of death in the United States [5]. To address the needs of patients with comorbid obesity and depression, a recent pragmatic clinical trial established the clinical effectiveness of treating both conditions together with an integrated collaborative care intervention called I-CARE (Integrated Coaching for Better Mood and Weight) [6].

I-CARE was a year-long program and combined 2 evidence-based approaches: the Group Lifestyle Balance (GLB) program for weight loss [7], which was adapted from the Diabetes Prevention Program [8], and the Program to Encourage Active Rewarding Lifestyles (PEARLS) for depression care management [9]. The Research Aimed at Improving both Mood and Weight (RAINBOW) trial demonstrated that, compared with usual care, the I-CARE intervention led to a significant reduction in body mass index (-0.7 [95% confidence interval (CI)]: -1.1 to -0.2); p = 0.01] and depressive symptoms (as measured by the Depressive Symptom Checklist (-0.2 [95% CI: -0.4 to 0.0)]; p = 0.01) over 12 months.

Germane to a type 1 effectiveness-implementation study design, the RAINBOW trial also included a longitudinal qualitative evaluation based on the 5 dimensions of the Reach, Effectiveness, Adoption, Implementation, and Maintenance (RE-AIM) [10] framework (Table 1) to understand I-CARE’s translational potential for wider uptake and dissemination in primary care for treating comorbid obesity and depression. We chose RE-AIM because it is an evaluation framework that had not been applied in a Hybrid Type 1 Trial [11]. However, the RE-AIM developers now call for its use as we have applied it here to study the RE-AIM dimensions early in program development [12]. Qualitative interviews were conducted with multiple stakeholders, including study participants, recruitment staff, intervention staff, and clinicians. The following research questions guided the longitudinal qualitative evaluation: (1) What are stakeholders’ perceptions along RE-AIM dimensions? (2) Do these stakeholders’ perspectives differ from each other? and (3) How do stakeholders’ perspectives change over the course of the study?

**Table 1:** RE-AIM dimensions and adaption in I-CARE.

Dimension	Definition	Adaptation in I-CARE
**Reach**	The number, percentage, or representativeness of participants in the study	The trial collected this information, probed what features of I-CARE enhanced the reach with the population and setting, and asked stakeholders how reach of I-CARE might be better achieved.
**Effectiveness**	Refers to the outcome effect of the intervention	The trial asked patients, clinicians, recruitment staff, and intervention staff how the intervention was having an impact and how effectiveness could be amplified.
**Adoption**	The number, percentage, or representativeness of intervention settings or stakeholders that would adopt the intervention	The trial asked stakeholders what they might recommend to other clinics that want to adopt I-CARE in the future.
**Implementation**	Refers to the fidelity of intervention delivery	The trial examined if participants reported using I-CARE strategies, stakeholder roles in implementing I-CARE, and stakeholders’ recommendations for improving future implementation of I-CARE.
**Maintenance**	Refers to the extent the intervention becomes institutionalized into routine practices and policies at adopting sites	The trial examined stakeholders’ perceptions of how to sustain the I-CARE program and maintain the program effect at the patient and primary care system levels in the long term.

## Methods

An overview of the interview focus for each stakeholder group and the timing of assessments for each RE-AIM dimension are shown in Table 2. We collected information at multiple time points to track how perspectives changed over time. For example, this allowed us to compare staff perspectives on how they planned to implement the program at the beginning of the study with what was actually implemented. The institutional review board for Sutter Health, Northern California, approved this study. Patients provided written consent, and other stakeholders did not provide consent as their participation was not considered human subjects research.

**Table 2:** Evaluation of RE-AIM dimensions, by timepoint and stakeholder group.

RE-AIM Dimension	Example questions	Months			
		0	6	12	24
**Participants**					
**Reach**	• When we described I-CARE to you during the informed consent process, what did you think about the time commitment involved?	●			
**Effectiveness**	• What do you think are the advantages of a program that addresses mood, physical activity, and weight together? What are the disadvantages? (*baseline)*	●	●	●	●
	• Which aspects of your care for mood, physical activity, and weight management are difficult to do? Which aspects are easy? (*follow-up)*				
	• How satisfied are you with the care you have received in the past 6 (or 12) months? (*follow-up)*				
**Adoption**	N/A				
**Implementation**	• What have you tried before to lose weight, be physically active, or help your mood? (*baseline*)	●	●	●	●
	• In the past 6 (or 12) months, what kind of care have you received to improve your mood, increase physical activity, and/or manage weight? (*follow-up)*				
**Maintenance**	• How likely are you to continue the strategies for managing your mood, physical activity, and weight that are part of this care?		●	●	●
**Recruitment Staff, Clinicians, and Intervention Staff**					
		**Baseline**	**Midpoint**		**Endpoint**
**Reach**	• What are the barriers you are experiencing in reaching your intended target population?	●	●		
	• How effective was reviewing the list of potentially eligible patients contained in the RAINBOW study packet for engaging PCPs in eligibility screening? (*baseline only)*				
**Effectiveness**	• What gaps, if any, can I-CARE fill in treating obesity and depression together?	●	●		●
	• What are the advantages of I-CARE’s integrated, team-based approach?				
	• How confident are you that I-CARE will achieve effectiveness across patient groups of different demographics and clinical characteristics?				
**Adoption**	• If I-CARE is successful, to what extent will other organizations like yours be interested in adopting this approach?	●	●		●
	• What do you think will be the greatest barriers to other PAMF sites or organizations adopting the I-CARE intervention?				
**Implementation**	• Describe your role and what you do in relation to the RAINBOW research study.	●	●		●
**Maintenance**	• What strategies or recommendations do you anticipate participants will continue after the I-CARE program?				●
	• If the I-CARE intervention were to be adopted by the healthcare system, what can be done to support the sustainability of the program?				

### Sampling strategy

In this article, “participant” refers to a patient who was enrolled in the main trial and who participated in the interviews. Among the main trial enrollees at baseline (prior to randomization), 10% were randomly selected for interviews. The patients were asked if they were willing to complete a short interview about their experience in the study and receive a $5 gift card. If the patient did not consent to the interview, the next participant was invited to take part in the interview.

Clinicians were chosen based on their role, availability, and willingness to participate. These included primary care providers (PCPs), chiefs/medical directors at the recruiting clinics, and physician advisors who served as consultants to the trial. All intervention and recruitment staff completed assessments. To respect the wishes of those who did not want to participate in the larger study (at the participant, intervention staff, and clinic staff levels), we did not ask them to complete an interview.

### Data collection

Semi-structured interviews were used for each stakeholder group and timepoint. Month 0 was the baseline assessment, Month 6 was the end of the intensive treatment phase, Month 12 was the end of the maintenance phase, and Month 24 was the end of treatment follow-up. We interviewed a total of 54 participants from the intervention group (n = 21) and control group (n = 33) in-person for 15 to 20 minutes after their study visit in their primary care clinic or by phone. Some, but not all, participants were interviewed at multiple time points due to lack of availability. Follow-up interviews with the control group included 14 participants who had dropped out of the intervention but agreed to continue participating in interviews. The number of interviews at each timepoint is summarized in Table 3.

**Table 3:** Summary of the number and types of participant interviews.

	Control Participants (N = 33)	Intervention Participants (N = 21)	All Participants (N = 54)
**Baseline pre-randomization**	N/A	N/A	47 (87%)
**End of intensive treatment phase**	17 (52%)	14 (67%)	31 (57%)
**End of maintenance phase**	15 (45%)	14 (67%)	29 (54%)
**End of treatment follow-up**	24 (73%)	11 (52%)	35 (65%)

PCPs were interviewed in group and individual formats for 30 minutes as follows. Three individual interviews (n = 2 at baseline; n = 1 at 24 months) and 8 group interviews were conducted during departmental meetings (n = 6 groups at baseline with 77 PCPs; n = 2 groups at 12 months with 34 PCPs); Ten 30- to 45-minute individual interviews were held with clinic chiefs/medical directors (n = 6 at baseline; n = 4 at 24 months), and four 30- to 45-minute individual interviews were held with MD advisors (n = 3 at baseline; n = 1 at 24 months). Intervention staff participated in eight 60-minute individual interviews (n = 3 at baseline; n = 3 at 12 months; n = 2 at 24 months). Input from study recruitment staff was collected via an online assessment that included open-ended questions related to their experiences (n = 3 at baseline; n = 3 at 12 months).

### I-CARE intervention

The trial protocol and main results have been described previously, including process variables such as intervention attendance, retention, and the intervention components delivered [6]. Briefly, the intervention combined 2 evidence-based interventions, GLB [7] for obesity and PEARLS [9] for depression, into a synergistic, year-long curriculum consisting of 2 phases. The intensive treatment phase included 9 individual face-to-face sessions of 60 minutes each (4 weekly, 2 biweekly, and then 3 monthly), and 11 home-viewed GLB videos of 20 to 30 minutes each, over 6 months. Participants received the PEARLS program for depression starting with the first in-person visit. The GLB video program started after the fifth intervention session. They were asked to record their weight, dietary intake, and minutes of leisure-time physical activity at least weekly using MyFitnessPal. They also were asked to wear a study-provided Fitbit pedometer that automatically uploaded daily steps into the participant’s Fitbit account. The maintenance phase included monthly 15- to 30-minute telephone sessions during months 6 through 12. A trained health coach delivered the intervention, reviewed participants’ self-reported data, monitored their progress, and used the data to facilitate individualized coaching during intervention sessions. The intervention team—including the health coach, the intervention manager, a psychiatrist, and a primary care physician—met biweekly to discuss participants’ progress.

### Analytic approach

Interviews were audio-recorded, transcribed, de-identified, and uploaded into NVivo 11. The coding team was led by the second author, a researcher with master’s-level training in public health and experience in qualitative analysis, under the supervision of the lead author, and with the assistance of 3 other master’s- and bachelor’s-level research assistants. The coding team was separate from the interviewing team and did not have any interaction or relationship with those interviewed. The Framework Analysis method [13] was applied, including (1) Familiarization—investigators reviewed transcripts to familiarize themselves with the data; (2) Identifying a thematic framework—investigators created a codebook to define key themes and concepts using the RE-AIM framework; (3) Indexing—investigators conducted coding trials to establish inter-rater reliability (IRR) and made revisions as needed until the trials achieved an acceptable IRR level (Cohen’s k = .75 or greater) for each code. Then, the remaining transcripts were coded independently; (4) Charting—investigators generated coding reports, summarized the data in coding memos, and developed a matrix of themes and illustrative quotes. Filler words—such as “um” and “like”—were removed from the quotes; and (5) Mapping and interpretation—investigators summarized the analysis across research questions and stakeholder groups.

## Results

### Participant characteristics

The demographic and health characteristics of the interview participants and the main trial participants are shown in Table 4. Overall, the demographic profile of interview participants is similar to that of the entire main trial sample. Most participants in this study were at least college educated (80%), non-Hispanic white (72%), and female (67%). Participants’ mean age was 54 years (SD 11.5), mean body mass index was 36.5 (SD 7.5), and mean score on the self-reported Symptom Checklist-20 depression scale (range 0 to 4) was 1.4 (SD 0.5). Retention in the main trial was higher among interview participants (89% to 94%) than in the entire RAINBOW sample (80% to 87%).

**Table 4:** Baseline demographic and health characteristics of interview and main trial participants.

Characteristic	Control (N = 33)	Intervention (N = 21)	All Interviewees (N = 54)	Entire RAINBOW Sample (N = 409)
**Age**				
18 ‒ <45	7 (21%)	4 (19%)	11 (20%)	115 (28%)
45 ‒ <65	20 (61%)	15 (71%)	35 (65%)	248 (61%)
65 ‒ <75	6 (18%)	2 (10%)	8 (15%)	42 (10%)
≥ 75	0 (0%)	0 (0%)	0 (0%)	4 (1%)
**Education level**				
High school graduate or less	0 (0%)	2 (10%)	2 (4%)	28 (7%)
Some college	5 (15%)	4 (19%)	9 (17%)	98 (24%)
College graduate	17 (52%)	4 (19%)	21 (39%)	150 (37%)
Post college	11 (33%)	11 (52%)	22 (41%)	133 (33%)
**Race/Ethnicity**				
Hispanic/Latino	3 (9%)	5 (24%)	8 (15%)	56 (14%)
Non-Hispanic White	27 (82%)	12 (57%)	39 (72%)	289 (71%)
Non-Hispanic Black	0 (0%)	0 (0%)	0 (0%)	6 (1%)
Asian/Pacific Islander	3 (9%)	2 (10%)	5 (9%)	40 (10%)
Other or refusal	0 (0%)	2 (10%)	2 (4%)	18 (4%)
**Sex**				
Male	10 (30%)	8 (38%)	18 (33%)	122 (30%)
Female	23 (70%)	13 (62%)	36 (67%)	287 (70%)
**Body mass index**				
27 ‒ <35	11 (33%)	4 (19%)	15 (28%)	209 (51%)
35 ‒ <40	7 (21%)	2 (10%)	9 (17%)	111 (27%)
40 ‒ <45	2 (6%)	9 (43%)	11 (20%)	44 (11%)
≥45	13 (39%)	6 (29%)	19 (35%)	45 (11%)
**SCL-20 ‒ Depression Symptom Checklist-20 (Range 0–4)**				
0 ‒ <2	31 (94%)	16 (76%)	47 (87%)	322 (79%)
2 ‒ <2.5	2 (6%)	5 (24%)	7 (13%)	76 (19%)
2.5 ‒ <3	0 (0%)	0 (0%)	0 (0%)	10 (2%)
≥3	0 (0%)	0 (0%)	0 (0%)	1 (<1%)
**Antidepressant medication**				
None	16 (48%)	10 (48%)	26 (48%)	240 (59%)
Paxil or Mirtazon	0 (0%)	0 (0%)	0 (0%)	4 (1%)
Wellbutrin	7 (21%)	6 (29%)	13 (24%)	42 (10%)
Other	10 (30%)	5 (24%)	15 (28%)	123 (30%)
**Number of hospitalizations within last 12 months**				
0	30 (91%)	19 (90%)	49 (91%)	374 (91%)
1	3 (9%)	2 (10%)	5 (9%)	31 (8%)
2	0 (0%)	0 (0%)	0 (0%)	3 (1%)
≥3	0 (0%)	0 (0%)	0 (0%)	1 (<1%)
**Retention in main trial**				
6-month assessment			51 (94%)	356 (87%)
12-month assessment			51 (94%)	344 (84%)
24-month assessment			48 (89%)	326 (80%)

### Results by RE-AIM dimension

The analysis yielded a range of themes identified for each RE-AIM dimension. In the narrative summary below, labels are used to correspond to these themes. We identified themes by RE-AIM dimension, which are summarized in Table 5; supporting comments for each theme are outlined in the, S1–S5 Tables. We collected and analyzed data from patients, recruitment staff, clinicians, and intervention staff separately. In the results below, we specify findings by group.

**Table 5:** Summary of themes across each RE-AIM dimension.

Dimension	Summary Themes
**Reach**	Time commitment (R1)
	Schedule flexibility (R2)
	Motivation for change (R3)
	Convenient location (R4)
	Health coach benefits (R5)
	Sensitivity and respect (R6)
	Recruitment methods to engage primary care providers (R7)
	Electronic health record integration (R8)
**Effectiveness**	Attitudes toward an integrated approach to addressing obesity and depression (E1)
	Variability in:
	Weight outcomes (E2)
	Physical activity outcomes (E3)
	Mood outcomes (E4)
	Advantages of intervention/reasons for satisfaction:
	Problem-solving and goal-setting skills (E5)
	Diet and exercise monitoring (E6)
	Health coaching (E7)
	Integrating exercise into daily life (E8)
	Disadvantages of intervention/reasons for dissatisfaction:
	Lack of individual tailoring (E9)
	Lack of accountability (E10)
	Effectiveness of team-based collaborative care (E11)
	Barriers and challenges to physical activity, mood, and weight management (E12)
**Adoption**	Return on investment and cost-effectiveness (A1)
	Physician buy-in (A2)
	Particular set of skills (A3)
	Operational resources (A4)
	Helps meet demand for psychiatric and preventive care in primary practice (A5)
**Implementation**	Engagement with and use of I-CARE components (I1)
	Coordination of medication management (I2)
**Maintenance**	Intent to continue strategies to manage weight and mood (M1)
	Confidence in ability to continue strategies to manage weight and mood (M2)
	Maintenance strategies (M3)
	Better mental health care needed for maintenance (M4)
	Ongoing staffing and resources needed to maintain program (M5)
	Health system role in supporting patients (M6)

#### Reach

Interviewers asked participants how the time commitment might impact their willingness to participate in a program like I-CARE (see, S1 Table, for supporting quotes for themes R1 through R8). Most participants did not consider time commitment to be a potential issue in participating in the study. Some participants stated that the time commitment needed for the program was a barrier, often because of work schedules or commuting (R1), and some worried about whether they could stay motivated or adhere to the program over the long term. However, many reported that the time commitment increased their willingness to participate because it meant the program was worthwhile (R3). Some participants mentioned facilitators to participation, such as having a flexible work schedule (R2), being motivated for change (R3), the program’s convenient location (R4), and the benefits of having a health coach (R5).

Recruitment staff also thought that time and scheduling were key factors impacting reach. Clinicians and intervention staff anticipated that the program would appeal to patients with time, energy, and resources, and that motivation and readiness to change would predict success. Clinicians also mentioned that stigma toward obesity and depression could influence reach in this population and emphasized the importance of respectfully approaching this sensitive topic (R6).

The trial optimized intervention reach by asking PCPs to review lists of potentially eligible patients and exclude those who would not be appropriate for the study because of medical reasons. Clinicians who reviewed the lists felt this strategy was beneficial (R7). For improving reach in the future, they recommended embedding the recruitment process into the electronic health record (EHR) (R8). They also suggested that doing outreach to staff in related disciplines, such as nutrition or diabetes education, could increase referrals to the program. Recruitment staff reported that the program’s reach was hindered by challenges common to many clinical trials, including having multiple exclusion criteria, patients being busy, patients not being responsive to calls or emails, and having out-of-date contact information.

#### Effectiveness

At baseline, most participants, intervention staff, and clinicians saw the promise of an integrated approach to addressing obesity and depression (E1) (see, S2 Table, for supporting quotes for themes E1 through E12). Yet, many participants also voiced concerns about this hybrid approach; the most common concern being the difficulty of addressing weight, physical activity, and mood at the same time, as these participants described:

“There are three ways to fail, I guess, that you may not move, you may overeat, and you may feel horrible all at once, as opposed to just trying to deal with one thing at a time.”

(Baseline participant)

“It could be that there will be a lot to keep track of and to do, and it might be easy to slack on one of those things and throw things out of whack that way…or to feel like if one of them is not improving, that you’re sort of failing the whole thing.”

(Baseline participant)

At the 12- and 24-month follow-up timepoints, some intervention participants articulated their personal experiences with how behavior changes to improve one outcome led to improvements in other primary outcomes and in secondary outcomes such as overall health, quality of life, and social functioning.

The findings showed evidence of within-group variation in treatment response. Intervention participants reported mixed results with weight (E2), physical activity (E3), and mood outcomes (E4). For weight, some participants said they had maintained but not lost weight; some said their weight had fluctuated greatly; some said they had lost weight; and some said they had gained weight. Some reported that the program had motivated them to exercise more and had made a large, sustainable improvement in their mood, such as this participant:

“I think that the first year, it put me on the right path, so I didn’t really have any major hiccups, I would say. I just kept on doing what I’ve been doing, and once I saw that it was not too bad, I was happy to live in it…. I keep walking because walking really is easy, and just, I think, extra time to walk even after work, carve out a little from the day and all the other stuff that I have to do, it just makes me feel better, and it feels like I’m doing something for myself, too, so it’s a good thing. Thank you.”

(Intervention participant, 24-month follow-up)

Others reported small or minimal improvement from the program, such as this participant:

“I’ve been doing this for a while, and nothing has really moved the needle significantly. My weight hasn’t changed much. I mean, I think my overall physical state is better if I do consistent cycle commuting…but I don’t have any heroic story about, ‘yeah, I lost 100 pounds.’”

(Intervention participant, 24-month follow-up)

Some reported facing challenges with health, psychosocial situations, or severe depression and that the program had not improved their weight, physical activity, or mood, such as this participant:

“It’s hard to lose weight…. The low motivation and the depression makes it hard to do anything, and just the cycle of having depressive episodes start back up again after me doing better is hard. It’s hard to find a good therapist, and I think that little behavioral interventions are not very helpful. They just leave the major issues intact, and they come back to get you later.”

(Intervention participant, 24-month follow-up)

Intervention staff corroborated the differences in outcomes reported by intervention participants (E4). They observed that the intervention was less effective for patients with severe depression or in difficult psychosocial situations than for patients without these challenges.

One way that the intervention may have improved outcomes for some participants was by addressing barriers to treating weight and mood. At baseline, participants reported that a lack of time, competing priorities, lack of motivation, and stress were key barriers to addressing their obesity and depression (E12). They also reported that their usual care providers did not provide enough accountability, counsel them on strategies to self-manage their mood and weight, help them set goals, or give helpful information (E12). Over time, many intervention participants reported that the intervention components helped them overcome these barriers. They said that problem- solving and goal-setting skills (E5), diet and exercise monitoring (E6), health coaching (E7), and building exercise into everyday life activities (E8) helped them address their weight, physical activity, and mood. In contrast, control group participants continued to report challenges with lack of time, stress, mood management, and adopting healthier behaviors throughout 6, 12, and 24 months, and most did not report using strategies similar to those taught in the intervention (E12).

Although many participants experienced positive outcomes in one or more areas, they had differing views about their satisfaction with the program, with about half being very satisfied and about half being very dissatisfied, including those who withdrew from the intervention (n = 14). Participants who were satisfied said this was because of improved outcomes (E2, E3, E4), gaining problem solving and goal setting skills (E5), self-monitoring (E6), and personalized individual health coaching (E7). Participants who were dissatisfied desired more individual tailoring because they felt the curriculum was not relevant to them, was too structured and impersonal, or was condescending (E9). To improve the effectiveness of the intervention, they suggested that it teach less information about the basics of nutrition and instead teach more practical guidance on implementing healthy habits. Participants also suggested that it include more health coaching support, such as a longer period of check-ins or peer support groups (E10).

In the view of clinicians and intervention staff, the way that the intervention may have improved outcomes was from the expertise provided from a team-based collaborative approach that contributed to program effectiveness (E11). Clinicians appreciated the extra support from the intervention staff and liked that their patients received more one-on-one attention.

#### Adoption

Clinicians and intervention staff were asked about barriers to adoption (see, S3 Table, for supporting quotes for themes A1 through A6). Clinicians and intervention staff stated that healthcare systems would likely be interested in the I-CARE intervention, but that it would be critical to show return on investment and cost-effectiveness (A1). With this type of evidence in hand, potential cost barriers related to insurance coverage or reimbursement for care would be easier to address, as these respondents described:

“Leadership will look at it and say, ‘okay, how this program is going?’ I know that, okay, we have good results, it’s effective, and all these things, but they also look carefully at the financial part of it and how it’s going to cover the cost-effectiveness…especially something that insurance is not going to cover.”

(MD Advisor, baseline)

“I think if the organization can see the rationale, see how much money that it’s actually saved, then maybe they would hire someone part time as a consulting psychiatrist, but they have to see the budget. They have to see the benefit of that in the budget.”

(Intervention Staff, baseline)

Through the midpoint and endpoint, clinicians and intervention staff continued to voice concerns about how this approach could be adopted and implemented as standard practice and incentivized because of the additional time required to deliver collaborative care. As these respondents explained:

“I think the team needs to be well-formed before and actually approach the primary care doctor and do a lot of education and [do] a lot of the organizational part, like organizing the workflow for the physician because I don’t think PCPs are able to have the time to start things like that.… I think there would be some sort of case manager within the primary care setting that could actually do outreach with patients and act as a liaison between the PCP and the team, and then the I-CARE team, obviously, but I do think there needs to be some support within the primary care setting.”

(Intervention Staff, 12-month follow-up)

“I think anything that requires more time on the physician’s side will be difficult to implement. I think teaching docs to do new things is difficult, too.”

(Chief/Medical Director, 24-month follow-up)

Physicians’ buy-in (A2) was another barrier clinicians and intervention staff identified at baseline and midpoint, as indicated by these respondents:

“It depends on…how much hassle physicians might perceive it to be if the communication across the team is time consuming. For some, there’ll be, I think, at least initially, a concern about loss of their sense of being autonomous or in charge.”

(MD Advisor, baseline)

“I think we’ve had some times where the PCPs don’t want to take our advice and things like that, and I think some of that is probably just the trust issue, because they don’t know us. They don’t really know who we are.”

(Intervention Staff, midpoint/12-month follow-up)

By the endpoint, they continued to recognize this barrier but had learned specific strategies to build buy-in, such as staff and departmental meetings. Key considerations they recommended as important for adoption included having an interdisciplinary team, building rapport and trust between PCPs and intervention staff, and getting buy-in across different clinics in a system.

“The primary care [provider] has to be willing to trust the health coach and to allocate some things and trust the psychiatrist.… If it were a new person coming in, if I were going to a clinic [where] I didn’t know the primary care doctors, I would want to meet with them, like weekly team meetings face-to-face, in the beginning at least, just to establish a rapport and a connection and trust.”

(Intervention Staff, 24-month follow-up)

“You’d have to come and talk to the doctors about it and see if they want to be involved in it, or if you want it to be implemented in primary care at a bigger level, I would say even discussing this at a department meeting—how everybody feels about it and at a bigger level than just one clinic level.”

(Chief/Medical Director, 24-month follow-up)

Finding a health coach with the specific set of integrative and collaborative care skills was also seen as a challenge by intervention staff at baseline (A3). At midpoint and endpoint, intervention staff continued to have concerns—and sometimes expressed even less confidence than before—about being able to train and retain a qualified, well-trained coach, and clinicians shared this concern after seeing how the program operated, as these respondents noted:

“I think the health coach actually has to have a very solid background in connecting with patients, having some level of counseling, plus having some global experience with dealing with depressed patients, anxious patients, and just their knowledge of health and nutrition, so I think attracting people that qualify can be difficult.”

(Intervention Staff, 12-month follow-up)

“It’s a different training than they have for a lot of our staff right now, and somebody that has experience [as a] health coach doing this, I think that’s more likely to have success. I’m trying to envision in the clinic who would do this and, in this format, the way you have it, I’m having trouble figuring out who would do this.”

(Chief/Medical Director, 24-month follow-up)

Clinicians and intervention staff also recognized the challenge of garnering the operational resources needed to integrate a collaborative care model into the workflow of busy primary care practices (A4). However, they believed workflow systems and policies would not be insurmountable barriers. By study end, intervention staff noted the need for more coordination support, including challenges with lack of clinic space.

Additionally, clinicians and intervention staff were asked about healthcare system interest in future adoption of the intervention. From the beginning to the end of the study, they consistently reported that adoption of I-CARE would help meet demand for psychiatric care and preventive care in primary practice settings (A5). They noted a key advantage of the team-based care approach that the I-CARE program offered was the alleviation of time pressure typically experienced in treating patients with comorbid obesity and depression, as these respondents stated:

“I think there would be a very high interest because this is an area …that we’re completely lacking, in terms of both weight management and mood disorder, so I think that it would be very welcome, especially if you can show that it has helped patients in the end result.”

(Chief/Medical Director, baseline)

“Most primary care doctors I deal with, I work with, would appreciate any form of help with mental health, because I think they’re just overwhelmed and overworked, so I think most primary care doctors would be open to it.”

(Intervention Staff, 12-month follow-up)

“All of our doctors are over-paneled, and I think we’re all a little bit short on time, right?. . .for weight management and depression. So I think it would be helpful.”

(MD Advisor, 24-month follow-up)

#### Implementation

At baseline, most patient stakeholders reported previous attempts to lose weight via physical activity, weight loss programs, and changes in dietary habits, and to treat depressive symptoms by seeing mental health professionals and using antidepressants (see, S4 Table, for supporting quotes for themes I1 through I2). Participants in the intervention group reported implementing one or more of the following I-CARE components (I1): exercise, food logging via MyFitnessPal, activity tracking via Fitbit, changing eating habits, and seeking self-help resources. Use of I-CARE components varied among participants and over time, in part because of the personalized nature of the coaching, which was tailored to participants’ goals and needs, but also because of individual differences in factors that could change over time, such as willingness to participate, physical and mental health, available time, stress, and competing priorities.

Several noted that their sessions with the coach revealed underlying issues about their mood and weight and formed a basis for working through these issues throughout 24 months. Participants in the control group reported using fewer resources to manage their weight and mood; primarily exercise, mental health professionals, and antidepressants. No consistent variations were found over time in strategies used by the control group participants, and they did not report the continued use of any strategies to the point of sustained improvement.

To implement I-CARE, the I-CARE team communicated with each other in regular meetings and collaborated with PCPs by telephone and staff messaging via EHRs. At baseline, some I-CARE staff felt challenged by not being fully integrated into the primary care clinics (I2). Also, clinicians raised concerns about the medication management workflow and potential delays in addressing patient needs. They recommended appointments be made automatically if the I-CARE psychiatrist suggested a change to the patient’s dosage and requested notes from I-CARE health coaches be placed in the EHR. Over time, clinicians reported that workflow concerns had been addressed through greater alignment and coordination among the I-CARE team and PCPs had improved and become more efficient (I2).

#### Maintenance

Participants were asked how likely they were to continue their strategies for managing weight, physical activity, and mood (see, S5 Table, for supporting quotes for themes M1 through M6). Throughout each follow-up timepoint at 6, 12, and 24 months, many intervention participants felt they were highly likely to continue or maintain their behavior changes, whereas control group participants often reported they were either not planning to make changes or were only considering behavior changes (M1).

Intervention participants varied in their level of confidence in maintaining behavior change at 6 months, whereas at the 12- and 24-month timepoints many reported they felt confident they could follow through, and had ways to handle challenges, such as restarting after a setback. In contrast, many control group participants had a consistently low level of confidence in their ability to manage weight and mood (M2).

Some control group participants also reported a desire to continue strategies to manage weight and mood, but also expressed challenges in making these changes, whereas intervention participants reported specific “SMART” (specific, measurable, attainable, relevant, and time-bound) strategies they would use to maintain successes made in the program (M3). For example, when talking about continuing strategies to manage their diet, intervention participants described several specific, doable steps, such as planning meals, measuring calories, scheduling activity, or finding small chunks of time to exercise. Control group participants tended to outline nonspecific goals, such as eating the right things or keeping physically active overall, and sometimes goals difficult to achieve, such as changing their entire routine.

When asked what the healthcare system could do to support them, several participants said that better mental healthcare would benefit them (M4). Other examples of the types of support desired included having a wellness coach available, follow-up from their PCP, and an “accountability buddy.”

At the end of treatment follow-up, clinical and intervention staff also offered concrete suggestions to support maintenance of the program, both at the patient- and the healthcare system-levels. They noted that adequacy of staffing would be key to maintaining the program over the long term, including dedicated coaches, low staff turnover, and coordinated care with physicians (M5). Clinical staff suggested that to support patients with weight and mood management, they needed to be able to monitor patients’ progress on weight, exercise, and mood, which could be enabled with preformatted templates in the EHR. The linchpin for a program like I-CARE would be to integrate it into the standard of care for patients who were managing both weight and mood (M6).

## Discussion

**Fig 1:**
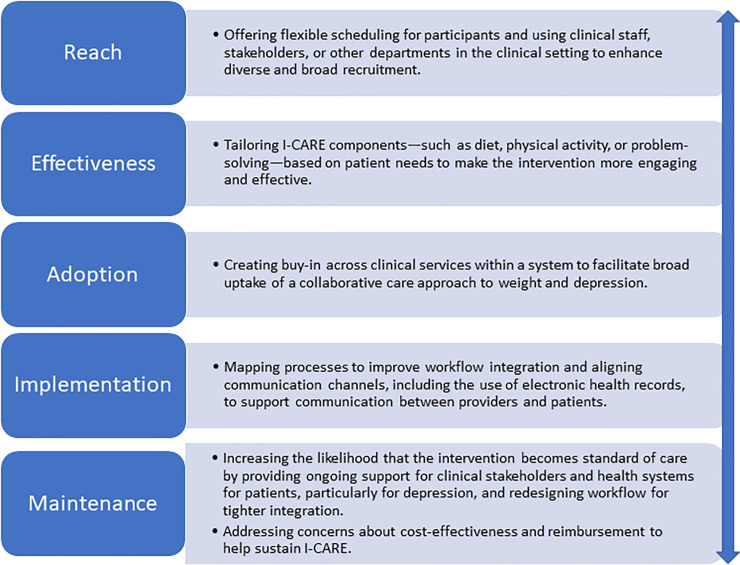
Key recommendations for improving I-CARE’s public health impact.

The present study used RE-AIM to understand how I-CARE’s translational potential for public health impact could be enhanced in subsequent implementations of the program. We examined multiple stakeholders’ perspectives along RE-AIM dimensions and whether these perspectives were similar and if they changed over time. Overall, at baseline, stakeholders expressed concerns about the ambitious nature of the intervention, but over time these concerns abated. Intervention participants clearly benefitted from receiving I-CARE compared to control participants. At the end of the intervention, stakeholders also shared positive beliefs about the potential for I-CARE to treat co-morbid depression and obesity. A summary of the key findings along RE-AIM dimensions is shown in Fig 1. Many of these recommendations reflect refinements for I-CARE in future iterations, such as tailoring to increase effectiveness. They also suggest implementation strategies that could provide a more supportive implementation context, such as aligning workflow and communication channels. Many of these recommendations likely strengthen multiple RE-AIM dimensions. RE-AIM developers acknowledge that these dimensions are not orthogonal.

The findings of this study shed light on how results from primary and secondary quantitative analyses from the RAINBOW trial may be improved in future iterations. For example, the main outcomes analysis revealed that 73.5% of intervention participants completed at least 7 of the 9 intensive treatment sessions, and 64.7% completed at least 5 of the 6 maintenance sessions [6]. Further, a priori secondary analyses revealed that (a) lifestyle and cognitive factors did not significantly mediate the main treatment effects as predicted [14], (b) sex significantly moderated treatment effects for both depression and weight, with males achieving greater reductions in BMI across multiple study time points and females achieving better depression at the 12-month timepoint [15], and (c) within-treatment analyses showed that poor engagement early in the intervention predicted treatment failure at the end of the intervention [16]. Future adaptations of I-CARE that increase the tailoring and targeting of intervention components to participants’ stated needs could also increase adherence and potentially enhance lifestyle and cognitive factors that mediate treatment effects by making I-CARE more engaging, thereby increasing effectiveness. A key translational issue to support these changes is the importance of coach training, support, and remediation of poor coaching. Further, economic analyses of the I-CARE intervention found that the intervention did not significantly increase annual spending on medical care services or antidepressant medications but increased appropriate antidepressant medication daily use [17]. Additionally, programs like I-CARE that successfully address multiple chronic conditions align with recent healthcare reforms in Medicare delivery and financing, such as changes to physician fee schedules and billing codes for more value-based chronic care management to improve population health [18–21]. These findings should alleviate health system stakeholders’ concerns.

This study provides three main methodological advances in applying RE-AIM. First, RE-AIM is not commonly used in the context of a clinical trial. It is more commonly applied with evidenced-based programs to quantify public health impact on program dissemination. Our application of RE-AIM in an earlier stage of evidence generation allowed us to quantify the *potential* public health impact and help future iterations of I-CARE achieve greater impact because the implementation was actively studied while effectiveness was being established. Second, multiple perspectives were obtained about I-CARE implementation over multiple time points. Incorporating multiple stakeholder perspectives—such as patient participants, I-CARE staff, and clinicians—is referred to as practice-based research. This approach is often overlooked, but it is one that is essential for designing the dissemination and future implementation and increasing the pragmatism of study findings [22]. Third, the study used qualitative methods. By listening to the voices of multiple stakeholders involved with I-CARE and probing their experiences and opinions over time, we were able to capture important themes aligned with RE-AIM dimensions that reflected stakeholder experiences. Recently, the originators of RE-AIM called for using qualitative methods and applying the framework more as a planning model, as we did in this study [12]; however, few have done so to date.

### Limitations

This study has limitations that condition our stated conclusions about the potential public health impact of I-CARE. First, although we randomly selected patient participants and included those who withdrew from the study, not all the selected participants completed an interview at each timepoint over the course of the study. However, in general, the demographic profile of participants in this study was similar to the overall demographic profile of participants in the main trial. Second, it was challenging to gain the time and attention of busy clinicians to obtain their input; consequently, interviews were more commonly conducted in a group format and some were conducted individually. Third, small numbers of clinicians and health system stakeholders were included in each role type, given their true roles in the health system in which the study took place. This means that some of the themes that emerged were based on small numbers of system stakeholders. However, for participants we had larger numbers and identified themes that emerged when numerous people’s input.

## Conclusions

Despite these limitations, using RE-AIM in this fashion helped provide guidance on how to increase the public health impact of I-CARE across RE-AIM dimensions. This is important because few evidenced-based approaches exist for treating comorbid obesity and depression. Speeding the translation of I-CARE and refining its effectiveness will help achieve the goals of integrating behavioral health in primary care [18]. Effective collaborative care approaches are needed to assist clinicians, support patients, and achieve population health.

## Supporting information

S1 Checklist Consolidated criteria for reporting qualitative studies (COREQ): 32-item checklist. (DOCX)

S1 Table Supporting quotes for the themes identified for the reach dimension^a^. ^a^Each quote is identified by the stakeholder type, stakeholder ID (if available), and timepoint. Condition assignment (intervention or control) is specified for participants at 6, 12, and 24 months, but not at baseline (pre-randomization). For participants, *baseline* refers to pre-randomization at enrollment, *6m* refers to the end of the intensive treatment phase (6 months after enrollment); *12m* refers to the end of the maintenance phase (12 months after enrollment); *24m* refers to the end of the treatment follow-up phase (24 months after enrollment). For other stakeholders, *baseline* refers to the beginning of trial; *12m* refers to 12 months after trial start; *24m* refers to the end of the trial.
(DOCX)

S2 Table Supporting quotes for the themes identified for the effectiveness dimension^a^. ^a^Each quote is identified by the stakeholder type, stakeholder ID (if available), and timepoint. Condition assignment (intervention or control) is specified for participants at 6, 12, and 24 months, but not at baseline (pre-randomization). For participants, *baseline* refers to pre-randomization at enrollment, *6m* refers to the end of the intensive treatment phase (6 months after enrollment); *12m* refers to the end of the maintenance phase (12 months after enrollment); *24m* refers to the end of the treatment follow-up phase (24 months after enrollment). For other stakeholders, *baseline* refers to the beginning of trial; *12m* refers to 12 months after trial start; *24m* refers to the end of the trial.
(DOCX)

S3 Table Supporting quotes for the themes identified for the adoption dimension^a^. ^a^Each quote is identified by the stakeholder type, stakeholder ID (if available), and timepoint. Condition assignment (intervention or control) is specified for participants at 6, 12, and 24 months, but not at baseline (pre-randomization). For participants, *baseline* refers to pre-randomization at enrollment, *6m* refers to the end of the intensive treatment phase (6 months after enrollment); *12m* refers to the end of the maintenance phase (12 months after enrollment); *24m* refers to the end of the treatment follow-up phase (24 months after enrollment). For other stakeholders, *baseline* refers to the beginning of trial; *12m* refers to 12 months after trial start; *24m* refers to the end of the trial.
(DOCX)

S4 Table Supporting quotes for the themes identified for the implementation dimension^a^. ^a^Each quote is identified by the stakeholder type, stakeholder ID (if available), and timepoint. Condition assignment (intervention or control) is specified for participants at 6, 12, and 24 months, but not at baseline (pre-randomization). For participants, *baseline* refers to pre-randomization at enrollment, *6m* refers to the end of the intensive treatment phase (6 months after enrollment); *12m* refers to the end of the maintenance phase (12 months after enrollment); *24m* refers to the end of the treatment follow-up phase (24 months after enrollment). For other stakeholders, *baseline* refers to the beginning of trial; *12m* refers to 12 months after trial start; *24m* refers to the end of the trial.* Note: PCP = Primary Care Provider.
(DOCX)

S5 Table Supporting quotes for the themes identified for the maintenance dimension^a^. ^a^Each quote is identified by the stakeholder type, stakeholder ID (if available), and timepoint. Condition assignment (intervention or control) is specified for participants at 6, 12, and 24 months, but not at baseline (pre-randomization). For participants, *baseline* refers to pre-randomization at enrollment, *6m* refers to the end of the intensive treatment phase (6 months after enrollment); *12m* refers to the end of the maintenance phase (12 months after enrollment); *24m* refers to the end of the treatment follow-up phase (24 months after enrollment). For other stakeholders, *baseline* refers to the beginning of trial; *12m* refers to 12 months after trial start; *24m* refers to the end of the trial.
(DOCX)
