# Supplementary material for: Using RE-AIM to examine the potential public health impact of an integrated collaborative care intervention for weight and depression management in primary care: Results from the RAINBOW trial
Source: PLoS One. 2021 Mar 11;16(3):e0248339. doi: 10.1371/journal.pone.0248339 (PMC7951877; doi:10.1371/journal.pone.0248339)
Supplement: S1 Checklist — (DOCX) [file pone.0248339.s001.docx]

Consolidated criteria for reporting qualitative studies (COREQ): 32-item checklist

| **No** | **Item** | **Guide questions/description** |
| --- | --- | --- |
| **Domain 1: Research team and reflexivity** |  |  |
| Personal Characteristics |  |  |
| 1. | Interviewer/facilitator | Which author/s conducted the interview or focus group? LRG and NL. They were assisted by three other research assistants who were MPH or Bas. |
| 2. | Credentials | What were the researcher's credentials? *E.g. PhD, MD* PhD, MPH, or BA |
| 3. | Occupation | What was their occupation at the time of the study? Researchers |
| 4. | Gender | Was the researcher male or female? Female |
| 5. | Experience and training | What experience or training did the researcher have?  Doctoral level training with experience in structured interviewing. |
| Relationship with participants |  |  |
| 6. | Relationship established | Was a relationship established prior to study commencement? No |
| 7. | Participant knowledge of the interviewer | What did the participants know about the researcher? e*.g. personal goals, reasons for doing the research* Participants were informed of the interview purpose in relationship to the main trial |
| 8. | Interviewer characteristics | What characteristics were reported about the interviewer/facilitator? e.g. *Bias, assumptions, reasons and interests in the research topic* None |
| **Domain 2: study design** |  |  |
| Theoretical framework |  |  |
| 9. | Methodological orientation and Theory | What methodological orientation was stated to underpin the study? RE-AIM |
| Participant selection |  |  |
| 10. | Sampling | How were participants selected? *e.g. purposive, convenience, consecutive, snowball* . Patient participants were chosen consecutively, and other stakeholders based on their role. (pages6-7) |
| 11. | Method of approach | How were participants approached? e*.g. face-to-face, telephone, mail, email*  In person (pages 6-7) |
| 12. | Sample size | How many participants were in the study? Varied based on stakeholder group. See pages 6-7. |
| 13. | Non-participation | How many people refused to participate or dropped out? Reasons? We retained many of those who dropped out of the main study in our analyses, but we do not know their reasons for dropping out. The completion rates for patient participants across time points are shown on page 7. |
| Setting |  |  |
| 14. | Setting of data collection | Where was the data collected? e*.g. home, clinic, workplace*  Interviews were conducted in the clinic or by phone based on interviewee preference to reduce participant burden. See page 7. |
| 15. | Presence of non-participants | Was anyone else present besides the participants and researchers? No |
| 16. | Description of sample | What are the important characteristics of the sample? *e.g. demographic data, date* See Table 3 page 9. |
| Data collection |  |  |
| 17. | Interview guide | Were questions, prompts, guides provided by the authors? Was it pilot tested? Yes |
| 18. | Repeat interviews | Were repeat interviews carried out? If yes, how many? Yes, see page 7. |
| 19. | Audio/visual recording | Did the research use audio or visual recording to collect the data? Audio recordings, see page 8 under Analytic Approach. |
| 20. | Field notes | Were field notes made during and/or after the interview or focus group? No |
| 21. | Duration | What was the duration of the interviews or focus group? Patient participants were 15-20 minutes, Clinicians 30 minutes, Clinic chiefs and medical directors 30-45, intervention staff 60 minutes. See page 7. |
| 22. | Data saturation | Was data saturation discussed? We used a priori N to guide the study |
| 23. | Transcripts returned | Were transcripts returned to participants for comment and/or correction? No |
| **Domain 3: analysis and findings** |  |  |
| Data analysis |  |  |
| 24. | Number of data coders | How many data coders coded the data? 3 |
| 25. | Description of the coding tree | Did authors provide a description of the coding tree? Our coding rubric and analysis approach is described on page 8-9. |
| 26. | Derivation of themes | Were themes identified in advance or derived from the data? Data derived based on RE-AIM dimensions. See Table 4. |
| 27. | Software | What software, if applicable, was used to manage the data? NVivo |
| 28. | Participant checking | Did participants provide feedback on the findings? No |
| Reporting |  |  |
| 29. | Quotations presented | Were participant quotations presented to illustrate the themes / findings? Was each quotation identified? e*.g. participant number* Yes, we include an extensive supplemental appendix for each RE-AIM dimension by stakeholder group by time. See supplemental appendices. |
| 30. | Data and findings consistent | Was there consistency between the data presented and the findings? Yes, see results and discussion sections. |
| 31. | Clarity of major themes | Were major themes clearly presented in the findings? Yes, see table 4. |
| 32. | Clarity of minor themes | Is there a description of diverse cases or discussion of minor themes? Yes, we highlight differing opinions across themes as relevant. |
