# Supplementary material for: Using RE-AIM to examine the potential public health impact of an integrated collaborative care intervention for weight and depression management in primary care: Results from the RAINBOW trial
Source: PLoS One. 2021 Mar 11;16(3):e0248339. doi: 10.1371/journal.pone.0248339 (PMC7951877; doi:10.1371/journal.pone.0248339)
Supplement: S1 Table — aEach quote is identified by the stakeholder type, stakeholder ID (if available), and timepoint. Condition assignment (intervention or control) is specified for participants at 6, 12, and 24 months, but not at baseline (pre-randomization). For participants, baseline refers to pre-randomization at enrollment, 6m refers to the end of the intensive treatment phase (6 months after enrollment); 12m refers to the end of the maintenance phase (12 months after enrollment); 24m refers to the end of the treatment follow-up phase (24 months after enrollment). For other stakeholders, baseline refers to the beginning of trial; 12m refers to 12 months after trial start; 24m refers to the end of the trial. (DOCX) [file pone.0248339.s002.docx]

**S1 Table. Supporting Quotes for Themes Identified for the Reach Dimension^1^**

| **Theme** | **Quote** |
| --- | --- |
| - 1. **Time commitment** | **Participants**   - - - “[The time commitment] is a little bit heavy, maybe, but not onerous. So, maybe a little bit more than I would expect from a research study but not too excessive. And especially given what we’re looking at and how it could theoretically help. For example, I can imagine that if I were in some weight management program with PAMF that I would have to put in at least that time commitment to do it. So, to me, it seems about in line with that.” *(MV08897, baseline)*     - “I think [the time commitment] will be mildly challenging just because I haven’t been in the habit of exercising in a formal way regularly. But if I’m picked, I would welcome it because I need that structure. […] It would be mildly challenging just because I’ve got two children and they’re on schedules, but I’m confident that I can make it work.” (*MV09434, baseline)*     - “[The time commitment] is a concern, basically, because my schedule during the week is so taken up, so I really don’t have much extra time to deal with. So, if I want to be part of the program, I have to make sure that either my work schedule will let me or I have to change certain things to make sure that I benefit from the program that I don’t waste the time of others and myself.” *(MV06639, baseline)*   **Recruitment Staff**   - - - ***“***People with inflexible jobs who feel they can't take away from work to attend visits. Even with our evening and weekend options, some who are interested just feel they don't have the time.” (*12m)* |
| - 1. **Schedule flexibility** | **Participants**   - - - “For me, it works out well, because I have recently retired, and I have time.” (*PA25965, baseline*)     - “I don’t think it’s unreasonable, and I don’t think it would be problematic for me, personally, just because there are evening appointments available. There’s flexibility in terms of the scheduling, so, yeah, it would work. It would work fine for me.” *(LA12164, baseline)*   **Recruitment Staff**   - - - “The retired age group are easier to reach by phone. They are also more flexible in their time to schedule appointments.” (*12m)* |
| - 1. **Motivation for change** | **Participants**   - - - “I think it's very reasonable in that if you want to get something out meaningful to have a result, you also have to be an active participant. If you're just an observer, then it doesn't cost you anything. Then you don't have any skin in the game.” (*PA28033, baseline)*     - “It depends on how determined you are to want to do it, because if I find that it is very important for my health, then I feel that I want to lose weight and I want to feel better, then I don’t mind spending more time. […] So, again, there is always give and take. […] To accomplish something, you have to put time and energy into it.” *(MV06639, baseline)*     - “It could be challenging, but I’m hoping that it actually produces some results. So, if it does, it would be really worthwhile. *(Interviewer: And to what extent did the level of time commitment to the iCare program influence your willingness to participate?)* So, I had to think about that. Time is the hardest thing to dig out. […] And I think it's lack of time that's causing a lot of my feelings of sadness. So, I think that it was difficult to decide to do it, but I think it might pay off. So that's what I’m hoping for.” (*PA22888, baseline)*   **Recruitment Staff**   - - - **“**Some potentially interested/eligible candidates have expressed reluctance to respond to recruitment outreach due to their reticence to make lifestyle changes.” (*baseline)* |
| - 1. **Convenient location** | **Participants**   - “It’s pretty close by. I work 15 minutes away at the most, so it’s easier for me to get here and do that kind of stuff.” *(PA21658, baseline)* - “It would be just a little extra effort because I come here a lot anyways. All my care team is here, so I have to walk one extra building.” *(LA12140, baseline)* |
| - 1. **Health coach benefits** | **Participants**   - - - “I know that it would be helpful for me. It seems like it would be for me, having the coach, because I really need someone to keep me accountable.” (*SU30700, baseline)*     - *“*I like the idea of having increased contact with somebody about it. […] I’ve certainly been trying to do this for a while by myself, and it’s not working, so I like the idea of somebody who is really at least somewhat more engaged than I have been by myself.” *(PA21658, baseline)* |
| - 1. **Sensitivity and respect** | **Clinical Staff**   - - - “I was in the room when it was introduced to my clinical department. […] People were busy saying, ‘what? You’re going to contact my patients and tell them they’re fat?’ So, I think it has to be done differently.” *(A02, baseline)*     - “There’s patients that [are] not ideal to call. A physician, the really obvious ones, will know. And, then I believe, there's a lot of projects happening, but I believe, I remember you had a very systematic way of going through and after we'd gone through that first screen, making sure later that they were appropriate to continue with that study, and that was very reassuring from a physician level […] I think [that] was good because of the sensitivity of the word ‘obesity and depression.’ […] I think that, from a physician's standpoint, was reassuring that we weren’t going to get a lot of complaint phone calls [laughing] (*Interviewer: So the multi-stage screening process was important to providers.*) And I think partly because of your topic. I think it’s less so with hypertension, it’s just not a taboo in that.” (*MD02, baseline)* |
| - 1. **Recruitment methods to engage primary care providers** | **Clinical Staff**   - - - “I think it's a really important step. I think there was a lot of fearfulness at our site about how much time does it take to review the list. So, a lot of the providers really thought that they would have to have to open the chart of everybody on the list, and that wasn’t the case at all, really. I think most people will know automatically that a patient is not going to qualify because of cancer.” *(PCP02, baseline)*     - **“**I felt like you did a very good job with the packet. […] I thought all that information was pretty clear.” *(A03, baseline)*     - “I think it's a good idea. Each doctor is going to be a little bit different. I know in my practice, I just basically said, ‘well, this is a good thing. I think everybody should at least know about it.’ So, I didn’t cross anybody off the list. But I do know that some docs are really particular on who they want other involvement with and so that at least gave them the opportunity, if they wanted to, to go through [and] cross people out. So, I think it's nice to have the option.” (*MD05, baseline)* |
| - 1. **Electronic health record (EHR) integration** | **Clinical Staff**   - - - “It’s a trade-off. When you give something in paper, it’s way easier to just quickly go through it. On the other hand, if you need to review a patient’s chart, obviously it’s a little more tedious than if you saw it electronically and could click. […] In a more ideal world, it would be delivered in EHR, I think, and then people could click in.” (*A02, baseline)*     - “The other thing [to recruit patients] will be to come up with some kind of dot phrases that explains the program and share it with the primary care physicians, so when they meet with a patient, they can put that dot phrase for the patient as educational , explain and put that dot phrase, that this is something that we offer with all the information, where does the patient need to contact, call, and all these things. […] That explains what the program is, where you offer it, how much does it cost, for example, covered by insurance or not, something like that for the patient.” *(A01, baseline)*     - “Perhaps you create some EHR basket or something where all of the outreach from the quality department or your department goes. Possibly that's something to consider so we can see what's going on. Otherwise, it's not part of [the patient’s] medical record and very difficult to field their questions.” (*MD01, baseline*) |
