# Supplementary material for: Using RE-AIM to examine the potential public health impact of an integrated collaborative care intervention for weight and depression management in primary care: Results from the RAINBOW trial
Source: PLoS One. 2021 Mar 11;16(3):e0248339. doi: 10.1371/journal.pone.0248339 (PMC7951877; doi:10.1371/journal.pone.0248339)
Supplement: S2 Table — aEach quote is identified by the stakeholder type, stakeholder ID (if available), and timepoint. Condition assignment (intervention or control) is specified for participants at 6, 12, and 24 months, but not at baseline (pre-randomization). For participants, baseline refers to pre-randomization at enrollment, 6m refers to the end of the intensive treatment phase (6 months after enrollment); 12m refers to the end of the maintenance phase (12 months after enrollment); 24m refers to the end of the treatment follow-up phase (24 months after enrollment). For other stakeholders, baseline refers to the beginning of trial; 12m refers to 12 months after trial start; 24m refers to the end of the trial. (DOCX) [file pone.0248339.s003.docx]

**S2 Table. Supporting Quotes for Themes Identified for the Effectiveness Dimension^a^**

| **Theme** | **Baseline** | **6m** | **12m** | **24m** |
| --- | --- | --- | --- | --- |
| **E1. Attitudes towards an integrated approach to addressing obesity and depression** | **Participants**:   - - “I strongly believe that having to work with mood and feelings and your diet have so much to go together, so if you address those issues, I'm sure that there's going to be a good outcome.” (*PA22725*)   - “I feel that they're all part of […] different stops of the cycle that all feed into each other. If one is out of whack, it gets the balance off.” (*PA26904*)   - “There are three ways to fail, I guess, that you may not move, you may over-eat, and you may feel horrible all at once, as opposed to just trying to deal with one thing at a time.” (*MV06084*)   - “It could be that there will be a lot to keep track of and to do, and it might be easy to slack on one of those things and throw things out of whack that way […] or to feel like if one of them is not improving, that you're sort of failing the whole thing.” (*MV08897*)   **Clinical Staff:**   - - “You’re deliberately tackling two conditions at the same time, and as we know for patients that have chronic illnesses, there is a huge mental health curve, and that sometimes goes unrecognized or untreated, so this [program], by connecting the two, is very, very powerful.” (*A03*) | **Intervention Group**   - *Minimal discussion of the connection between exercise, mood, and weight. Discussion was focused on specific behavior changes.*   **Control Group**   - “I don’t know what the counselors are doing in this study, but it would be nice if somebody was holistic, if they had like a more holistic approach, if they were able to talk about balancing the three things. They’re obviously related, so that would’ve been interesting, yeah, and I can say, it might have been helpful.” (*PA22014*) | **Intervention Group**   - - “I would never have associated the connection between that exercise we’ve just been talking about and one’s mental well-being if it wasn’t for this program. I wouldn’t have thought of it, and I can distinctly see that there is a connection.” (*PA27047*) | **Intervention Group**   - - “It was good because it kept weight loss in the forefront of my mind and getting more activity in at the forefront and also the emotional issues, too.” (*LA11670*)   - “I carved out a little time for myself before going home. I make an additional walk before I go to the bus station. That also improves the amount I move or the steps I take every day and makes me sleep better, makes me more relaxed to come home, so this extra 10, 15 minutes’ walk that I make for myself really improves both keeping off the extra weight and feeling better.” (*MV06639*)   **Intervention Staff**   - - “I see with the increase in preventative medicine and trying to get people to be healthier and lose weight […] I think this program can be adopted. […] It does have its successes. It does address a lot of the mental health aspects or the mood aspects of a participant. It helps to increase the length that that they’ll be able to adopt the lifestyle changes.” (*I04*) |
| **E2. Variability in weight outcomes** | **Participants**   - “As far as weight goes, I've known for a long time that the only way I can lose weight is through exercise, and so, the fitness center was my usual go-to place […] but it's not possible based on my current workplace.” (*MV06084*) - “For losing the weight, it is, I guess just being, the hardest thing is being honest with myself.” (*PA24005*) - “Even when I tried to reduce calories and move a little more, I never saw any changes in my weight, so not to see any result or not to know how long I have to wait to see result was very discouraging.” (*MV06639*) | **Intervention Group**   - “When I started this program, I was actually gaining weight, and I don't think I’ve gained since I started. I don't think I’ve ever actually lost, but still.” (*MV06084*) - “[I-CARE] has been helpful. It's been slow progress, but I think it's been good. And, yeah, the weight has been slowly, I have increased my physical activity, although I was pretty active before. And so physical activity for me is not really a problem. It was the, I just wasn't making any progress losing weight and plateaued for a while in the middle of it. But that helped.” (*PA24005*) - “I am very satisfied. I got a lot of help resolving issues. I also got help on finding time to exercise, and the first time in the last five or so years, I am finally losing all the weight, which really makes me very happy.” (*MV06639*) - “I had been being good and recording everything, and I was within all the parameters and not eating and putting out more energy than I’m putting in, and still barely losing any weight at all. It was like, “what's the point of all this effort if it's not doing anything?” (*MV09434*)   **Control Group**   - “For a period of time, I was very energized, and so I had a lot of energy to walk and to do more, and so I lost a little bit of weight, but then I put it back on, so that was unfortunate. That was about maybe a month or so ago. I lost the weight, and then it came back.” (*PA22725*) | **Intervention Group**   - “I think I can pretty much maintain [my weight]. I just can’t lose it.” (*MV06084*) - “It’s been down, and it’s been up and down. I was doing pretty well until the summer, and then after the summer, it’s starting to go back up again this last month or two.” (*PA24005*) - “I had some significant ups and downs with weight, I think because of the drug prednisone, which I was on temporarily. […] But I haven’t been able to get my weight down, and so my weight before I came on the program was in or about 220 pounds, and it’s now 230 pounds, and I really want to get that down, but I don’t have the willpower to do what [the coach] tells me to do, which is to stop eating between meals and not to buy some kinds of stuff in the supermarket.” (*PA27047*)   **Control Group**   - “Any challenges? Well, yes. I would say that I haven't seen my weight go down.” (*MV01109*) - “In terms of weight, knowing how to do is—it's a question of eat less and exercise more. Calories out, calories in as you lose weight. But yeah, actually doing that—I have not been successful.” (*MV03729*) | **Intervention Group**   - “There were a lot other pretty awful things happening to me. So, concerns about weight were — even though I wasn’t actively gaining—they were somewhat secondary to all the other things that they were doing.” (*MV06084*) - “Actually losing some weight is a challenge. I lose some and then gain it back, and then I lose some and gain it back. I never seem to get out of it.” (*PA24005*) - “In the weekend, looking at the weight and [I] said, ‘okay, I didn't gain any, so I must be doing something right.’” (*MV06639*) - “I think that the program has spurred me to lose the weight, to start focusing on writing down things and monitoring my exercise, so, yeah, it’s been successful because I’ve lost about 30 pounds.” (*MV01332*)   **Control Group**   - “[I’m] not very satisfied because instead of losing weight, I think I gained a lot more. But I think it had a lot to do with my depression and my mood, because last year around this time, that's when—yeah, at the end of May—that's when I lost my job. And so my depression plummeted. Yeah, my mood plummeted, and so, yeah, from then on things went downhill.” (*PA22725*) - “I want to do more, but unfortunately, I just can’t. I had my torn foot muscle and that really pushed me back in regards to my goal weight, and unfortunately, I’m still dealing with that.” (*SU33350*) |
| **E3. Variability in physical activity outcomes** | **Participants**   - “My schedule during the week is so taken up, so I really don’t have much extra time to deal with.” (*MV06639*) - “If I have 8am meetings every morning and 8-6 meetings, I don't have time to exercise or I'm too tired to exercise. If some of it is, I can imagine encouragement, and reminders are helpful. But I've tried to do a lot of those things myself. My watch will tell me to stand up and go walk around, right? So, how much additional, this sort of thing to address that, I don't know. It remains to be seen.” (*PA21152*) | **Intervention Group**   - “The physical activity is hard, because I work eight hours. I spend three hours just commuting daily. I am not a young lady anymore, so when I get home at night, I am usually exhausted, and the last thing on my mind is to do some exercise.” (*MV06639*) - “[The program] didn’t change me a lot because I already tend to do a lot of exercise and I have a lot of outside programs I do through my work […] so I was already doing a lot of that stuff, and I don't feel the course actually changed a lot.” (*PA21152*) - “I spend a lot of time with socializing, and the socializing is with friends that like to go out and walk. […] I’ve changed up the things that I’ve been doing versus just sitting at home and watching TV.” (*MV03987*)   **Control Group:**   - “I think motivation is an issue. I’m more likely to do the classes or something if they’re at a time when it fits into my schedule, so, for example, if I go home after work, it’s harder to be motivated to go back out at 8:00 for a class.” (*MV00993*) - **“**I’m out of breath. […] I’ve taken a couple of shots, playing some basketball, and just shooting, running around or running outside, you have to go to the garage or something like that. I can feel like I’m not where I should be, so I just don’t do anything.” (*PA30091*) | **Intervention Group**   - “I really did work quite a bit, and it really helped my mood and sleep and everything, the additional exercise.” (*MV06639*) - “The exercise, well, I had been set up to do regular cycling anyway. […]. The one thing it did provide me was a reason for, you know, when, in the morning, you go, ‘gosh, I don’t feel like riding to work,’ it says, ‘no, you're going to ride to work,’ so motivation was helpful.” (*PA21152*) - “I started working out at a gym, doing a lot more walking than I had been doing previously. I’ve been working out with a partner and spending more time, believe it or not, outdoors in the open, which I think has a huge effect on our mental state. […] I’ve been very satisfied with it. It’s provided me with some direction and put some concrete foundation down for me. […] I think it’s really the physical activity. It’s really been a boost for me.” (*MV03987*) - “I’m in the program with the coach, but unfortunately I literally have not done anything. My daughter had heart surgery so it was very busy. […] It’s just been the stress of our situation.” (*MV11150*)   **Control Group**   - “Pain prevents me from doing almost any exercise. […] When I'm well enough to do any exercise, what I end up doing is house chores, because they need to get done.” (*MV05164*) - “I’m just, I’m tired at the end of the day. I used to be able to walk afterwards. And now, even the dog, walking the dog is very difficult for me to even do that, to take him for a walk. I feel bad that I’m not able.” (*PA22725*) | **Intervention Group**   - “I think that the first year, it put me on the right path, so I didn't really have any major hiccups, I would say. I just kept on doing what I’ve been doing, and once I saw that it was not too bad, I was happy to live in it. […] I keep walking because walking really is easy, and just, I think, extra time to walk even after work, carve out a little from the day and all the other stuff that I have to do, it just makes me feel better, and it feels like I’m doing something for myself, too, so it's a good thing. Thank you.” (*MV06639*) - “I’ve been doing this for a while, and nothing has really moved the needle significantly. My weight hasn’t changed much. I mean, I think my overall physical state is better if I do consistent cycle commuting […] but I don’t have any heroic story about, ‘yeah, I lost 100 pounds.’”(*PA21152*) - “Over the course of time, I’ve had trouble with one of my knees, so I’ve got to get that taken care of, but that’s limited my activity, basically—where I used to do a lot of walking or hiking, I’m now down to just very limited amounts of time […] So that’s really impacted.” (*MV03987*)   **Control Group**   - “I tried and failed to sign up for a yoga thing. I downloaded an app, and I never did anything about it. And I’ve been pretty much sick, so I haven’t done much of anything else. I had been unable to—my best exercise is to walk outside, and I’ve been totally unable to do that.” (*MV05164*) |
| **E4. Variability in mood outcomes** | **Participants**   - **“**The expectations [about I-CARE] would be to get myself back in shape, to feel better about myself, because right now I don’t feel good about myself, of definitely trying to look nice, trying to go out, trying to do things with, especially now with my grandson.” (*MV10489*)   **Intervention Staff**   - **“**I think that we're going to be somewhat effective in depression, especially in getting people who weren't very well treated on to a better path. And with the behavioral skills, and adjusting medications, I think we'll be fairly effective there.” (*I02*) | **Intervention Group**   - “Through the help with [the coach], he's helped me to see these things in a different perspective and work out some of the emotional feelings that I have carried for two years.” (*MV10489*) - “[Mood] is an ongoing thing. I mean, I struggle with depression. It's terrible, and so the only thing that I do, that I have done for that, now what can I say? Standard sort of stuff. I mean, I make sure that I get out.” (*PA26094*) - “I haven't found the mood portion of it to be particularly helpful, and that may have more to say with where I am on that continuum than anything else, but I found the whole worksheet thing to be kind of, it’s not very, it wouldn’t be, it doesn't seem like something that would help to address any serious problems I have.” (*PA21152*)   **Control Group**   - “I don’t want my whole life to be in mental health treatment. I want to get well. I’m tired of this. I’m tired of being sad sometimes and all that. I’m tired of being lonely. I have walls about relationships, and I don’t trust people. Abandonment. I’ve got all kinds of issues. […] When I’m having symptoms, I’m embarrassed—not embarrassed, but I don’t want people to see me like that, so I cocoon. I stay home. I don’t go, I don’t really have anybody that I could reach out to for help.” (*LA12140*) | **Intervention Group**   - “When I came into the program, my mood, I had had two deaths in the family, so I was carrying a lot of—not guilt, but loss—and so after doing […] the questionnaire every time, it really did help my mood and mindset.” (*MV10489*) - “I think what was easy for me is the portion about the mood. It was easy for me to work from being a couch potato then going out and finding activities to do, whether it was going out with a friend to take a walk or going to spend time reading. […] Before I wouldn’t do that. I even do that if I’m alone now. That has really boosted some positive vibes for me.” (*MV03987*) - “I've had a lot of depression, and that's been the low energy that came with it, or maybe low energy provoked or prompted the depression. I don't know which is which. Part of that is my sleep schedule is fairly bad still. […] I’m not sure how I could change that, at least not through the medical facility.” (*PA30878*)   **Control Group**   - “I’ve been receiving psychiatric care. […] So I’m taking more medication to help me stabilize my mood—Wellbutrin, Lexapro, Neurontin, and one more.” (*PA22725*) - “Improving my mood's always been a challenge. That's why I'm not working, because if I could improve my mood, I would just improve my mood, and I could go work.” (*LA12140*) | **Intervention Group:**   - “This program, which I thought was going to help me more with my weight, it actually helped me more with my state of mind and mood and realizing there’s other things. For just weight, like I said, I really didn’t lose that much.” (*MV10489*) - “It’s hard to lose weight […] The low motivation and the depression makes it hard to do anything, and just the cycle of having depressive episodes start back up again after me doing better is hard. It’s hard to find a good therapist, and I think that little behavioral interventions are not very helpful. They just leave the major issues intact, and they come back to get you later.” (*MV04933*)   **Control Group**   - “I’m really having a hard time, even going to appointments is hard for me. That’s why I stopped going to the physical therapy, it’s just, I just dreaded going. I felt good after I went, but I just hated going. I’d get so much anxiety. And I don’t know why.” (*LA12140*)   **Intervention Staff**   - “I think I-CARE could be effective for a really broad range of populations. I do think maybe there are some treatment resistant depression cases where patients have tried multiple meds and the primary care physician may not feel comfortable trying a fifth medication. They might want to see a psychiatrist, which is reasonable.” (*I03*) - “It depends on the severity of the depression. I found that that plays a big role as far as how successful they are in the program. […] For more severe depression, I think that’s going to take more of treatment and care through psychiatry as well as their physicians, and once their major issues are addressed, then coming back to the program after that. ” (*I04*) |
| **E5. Advantages of intervention/reasons for satisfaction: problem-solving and goal-setting skills** | **Participants**   - “I have a habit of doing things really strong for a few weeks and then for some reason backing off. I don’t know why, I just—and then a few weeks will go by and I’ll re-motivate and engage for a couple weeks and then I’ll lose focus, and I don’t know why.” (*PA21735*) - “Maybe [the health coach] has a repertoire of avenues to approach different problems that are beyond things I've tried for myself. And, so that, I can see that that could be very helpful.” *(PA26904)* | **Intervention Group**   - “It’s been helpful to me and good. They make me be much more specific about my goals […] which I think is making me more successful in my weight loss. […] I feel like I’ve solved a good chunk of problems, rather than just focus on the same one, lose weight all the time. That's not a good goal, so [this is] much more broken down.” *(PA21735)* - “I had purchased some trekking poles, and I hadn't put them together. I hadn't taken them out of the box, and so the [goal] was to get them out of the box and start using them. And then, ‘how far do you think you can walk?’ and setting a goal and all that sort of stuff. And it just was the greatest thing that happened, and I was out there every morning, walking further and further and further.” (*PA26904)* | **Intervention Group**   - “[The coach and I] solved problems together [about] what was bothering me or what I wanted to accomplish. And then we came up with solutions, A, B, C. ‘What would be a good outcome? What would be the better outcome? What if you didn't do anything?’ So it helped me to not to stress out so much about every little thing in life that came in my way.” *(MV06639)* | **Intervention Group**   - “I do have mood swings, that in the past would set me off, and eating was how I would deal with it. I don’t now because of some of the classes I’ve had and some inputs that I’ve had. I was with the individual [health coach]. I really liked that. We set up goals and what to do after them. It really helps with my mood.” *(PA26904)* |
| **E6. Advantages of intervention/reasons for satisfaction: diet and exercise monitoring** | **Participants**   - “I’m starting to exercise again, but probably having a plan for doing more than just walking, a lack of a plan or whatever.” (*MV01332*) | **Intervention Group**   - “I think that logging it in the long run and just forcing myself to do it, it makes me more aware of how many calories I’m eating, and then it’s easier, a little bit easier for me in my head to know, ‘okay, you have 500 calories more.’” (*MV01332*) - “[My Fitness Pal] has trained me how to spread out 1,200 calories and stick to it. And then at the end when you sign off, it says, ‘if you keep it up, in five weeks, you’ll weigh this much,’ and you’re like, ‘oh, that's cool.’ So the little things that I get from that app is all I need. It's perfect, and it's more than I’ve ever had before, so it's made a difference.” *(PA21735)* - “I want to say that I think the most clever thing that you people have done is the Fit Bit. I think that, in a nutshell, itself helped keep me in as long as it did. Because it's like you have a little pal with you all the time, and that pal tells you how you've been doing. And so that's very, very helpful.” (*PA26904*) | **Intervention Group**   - “I've been using MyFitnessPal, and that is the most awesome app. It's very motivating. It sends you e-mails, ‘here's what you're doing.’ I'm competing with my friend. Every week we get an e-mail. That's been fun. I get a kick out of closing out every night. I think that's helped me. […] I'm going to keep using it more because it helps me change my patterns.” *(PA21735)* - “I also am happy wearing this device that tells me how many steps I took a day. It really helps me to feel good about the little changes I am doing—so instead of taking the elevator, going down the stairs*.” (MV06639)* | **Intervention Group**   - “I’m also paying attention more to my Fitbit. It reminds to get up and move, and before I wasn’t paying attention to it, but now I’m trying to pay attention to that just to get up.” *(MV01332)* - “I would say that the last year was no different really than the year before. I just kept doing the same things, and I really relied on the app to write down stuff. And then it would say, ‘oh, you only have 100 more calories left for the whole rest of the day.’ It kind of kept me honest. And then I use my Fitbit. So I’d say, just being vigilant with those two.” *(PA21735)* - “It's a manageable level of nuisance to do, and it helps to be aware of what you're eating and sometimes helps you to decide whether you can actually afford—from a calorie point of view—to eat that.” *(SU33803)* |
| **E7. Advantages of intervention/reasons for satisfaction: health coaching** | **Participants**   - “I would hope that the coach would have some ideas as to how I can address the portion control and even how I can be motivated.” *(PA27047)* | **Intervention Group**   - “I’ve valued the opportunity to talk to [the health coach] across the table. Because whatever chance I have of doing things that I don't particularly like doing, I have a much better chance if I make a commitment to him in real life rather than over a screen. I just think availability, as it were, is very valuable. I think it's a clever program the way you organized it. So, you have somebody at the beginning to walk you through the thing and answer your questions and so on and then you can take it to the phone. It's easier to do if you know the person at the other end of the phone.” *(PA27047)* - “I’m going to have to give [the program] an A+ for me because I was very sedentary six months ago. And I think just the simple fact that somebody paid attention to me, that was [concerned with intervening] with my weight and my mood, really taught me a lesson there, that I can’t just be complacent and just keep doing the same thing all the time.” *(MV03987)* | **Intervention Group**   - “With the coach telling me that I am doing okay and he is pleased with me, then it really helps me to stay focused, stay in the program, stay positive about even the little achievement I could come up with.” *(MV06639)* | **Intervention Group**   - “I think it’s all been more beneficial when you have someone with you as opposed to doing it all by yourself.” *(PA23283)* - “As begrudging as the one-on-ones were, I still had his voice echoing in my head and those exercises of planning it out, what could happen, what could go wrong. So I would remember the deals that I made, like, ‘don’t go into the kitchen after 8:00 at night.’” *(PA21735)* |
| **E8. Advantages of intervention/reasons for satisfaction: integrating exercise into daily life** | **Participants**   - “It's hard to focus on one thing and try to connect my emotions with everything else, I mean, it's just a disconnect for me. […] And a lot going on, and I think that's what it is—being busy. But it's part of my life because I have family, I have work.” (*SU33352*) - “I think with those other things that I’ve done before was as much as, I mean, I did it, I got to where I wanted, but then I didn’t know how to manage it from there. I didn’t know how to incorporate real life with it.” (*PA29161*) | **Intervention Group**   - “I belong to a gym, but it's extremely inconvenient for me because I take the train to work. […] So that’s, like I said, I’m very careful to do a lot of walking.” (*MV06084*) | **Intervention Group**   - “I haven’t joined a gym, but there were several issues that came to with being sick and with my knee, so it was kind of limited, but those were the type of activities that I’ve done within the last six months, just going out of the house and walking back and forth in different areas, communities, and malls. And I try to do every errand that I need to do as part of an activity deal for me.” (*SU33352*) - “Cycling to work was great for me because I like cycling, and I need to go to work anyway, and it's only a few minutes longer. It only takes a few extra minutes out of my day. And so there’s a lot of advantages on both sides of the fence. So this is a thing I can do without having to stress a lot about it.” *(PA21152)* | **Intervention Group**   - “We just started Little League baseball, and it’s funny. We find ourselves out there with [our son] in the practice. He’s only been going a couple weeks, but you find yourself being more active out there because he’s out there, and you’re practicing with him.” *(PA29161*) |
| **E9. Disadvantages of intervention/reasons for dissatisfaction: lack of individual tailoring** | **Participants**   - “I-CARE is making the assumption that you are starting at the beginning, and most of us who have weight are never starting at the beginning. We've done many of these things before. So, it's not that we don't know what to do. It's just that we haven't found the right combination to do it yet.” *(PA30878)* | **Intervention Group**   - “The focus was all for pre-diabetic people. I'm not pre-diabetic. […] If the individual did not have any prior knowledge of nutrition, diet, body chemistry, any of that sort of thing, it would be a really good program, but that's not me.” *(PA26904)* - “Every time, [the coach] has the same canned response and it just seems really not authentic. […] I think a coach should want to know some about you and your situation and get to know you as a person. […] He’s just filtered, like I’m talking to a robot.” *(SU30700)* | **Intervention Group**   - “[The coach] used to review the recorded sessions with a bunch of shrinks, and they seem very quick to recommend medication. […] The medication seems to be offered just because I hit that threshold, even though […] I have a history of medication side effects.” *(MV06084)* - “Not only do I not feel like it's helping me, I don’t even feel like it's trying to help me. The program is so structured. […] It isn’t tailored to me in any particular way. […] I’d show up for 15 minutes, and we’d walk through the binder, right? So it's like going to the DMV.” *(PA21152)* | **Intervention Group**   - “It’s actually a little condescending to immediately put someone my size into a nutrition program or something like that, or tell them to go see the nutritionist, because it’s not like somebody who’s lived with their weight all their life doesn’t know what nutrition is or how to deal with it.” *(PA30878)* - “It’s finding people who recognize that not everybody is at the beginning, you know? Meeting people where they are and taking them where they need to go, rather than starting everybody here, this doesn’t work**.** […] Just because we know it, we don’t know how to use it or we don’t know how to implement it in our lives, and that’s what we need help with.” *(MV04106)* - “I found the videos at too low a level of thought process. Something deemed a college graduate [level] would have been more effective for me.” *(SU33803)* |
| **E10. Disadvantages of intervention/reasons for dissatisfaction: lack of accountability** | **Participants**  “I’m hoping it’s the coaching piece [that could help me], and I’m hoping that it would be sharing of information and knowledge that if you do this, this could be the improvement. Or if you don’t do this, you’ll continue to struggle in this area. So I’m hoping that the expertise from the program, whatever’s learned from the coaches, and what they’ve learned from other participants can help me out.” *(MV03987)* | **Intervention Group**   - “I think the only benefit I could get from a coaching experience is somebody who's on my ass. And I'm not sure that exists here either because it's just like, ‘Okay, you had the cheesecake, don't do that next time.’” *(PA21152)* | **Intervention Group**   - “[The maintenance treatment phase] was too spaced out in a way. […] It’s that checking in with someone, I think, that was helpful. […] A phone call every other week or […] maybe even an email would help because you had to write it down, because I wasn’t good at logging. […] Just that weekly check-in to say, ‘yes, I did this much exercise, I tried to stick to this, or I had this problem.’” *(PA24005)* | **Intervention Group**   - “It helps me to engage better if I’ve got somebody there. I think the tough thing for me is I wasn’t part of group where I can absorb information from others or get inspiration from others. […] It would be terrific even to have a 30-minute online check-in once a month with different people, folks were in the same situation in life as I am, let’s say. They’re working, and we’re struggling with weight or whatever it be, or our health.” *(MV03987)* |
| **E11. Effectiveness of team-based collaborative care** | **Clinical Staff**   - “You have this health coach who's saying, ‘okay, this is a list of people we're looking at. These are the scores.’ It’s systematically addressed, whereas in primary care, it feels more like a free-for-all.” *(I03)* - “The idea that there is a health coach available, because patients are going to run into challenges and barriers and help with problem solving them in ways that are agnostic to the condition, to me is very, very helpful.” *(A02)* - “[The health coaches] have the time to sit longer than 15 minutes and get into the details about what's going on at home, because ultimately, that's probably the biggest issue […] and we don’t have that time to do that.” *(MD05)* | n/a | n/a | **Intervention Staff**   - “I think having that team approach, especially with patients […] they can hear multiple different perspectives and how their depression can be treated in multiple different ways. And also just getting that reinforcement that, yes, three or four professionals in different fields feel like you may be depressed and you could benefit from these different strategies. So I think more people buy into it more when there’s more of a team approach, actually.” (*I03*) - “The field of medicine has become so specialized. So, I think in our team approach, we actually can see a bigger picture [by] reviewing a patient's chart together. […] It’s giving more a global view of the patient.” *(I03)* |
| **E12. Barriers and challenges to physical activity, mood, and weight management** | **Participants**  ***Lack of time***   - “Barriers for physical activity is, time commitments, life gets in the way of stuff, motivation can be a problem, injuries, right? Same thing with dieting, you'll hit into the Christmas season and, or you get depressed and just start shoving food into your face of something like that. Like I said, I haven't done a lot to try to do mood. Some of that is lack of motivation to actually do something about it.” (*PA21152*) - “I can usually initiate something, but then life and time gets in the way. Lack of time is the most important thing. Feeling too busy, and it just stops whatever I try. I just resort to going back to just doing nothing.” (*PA22888*)   ***Stress***   - “I'd probably say the biggest barrier would be stress, and stress level with my depression and anxiety creates sort of lower frustration tolerance for me. And basically, the more stresses that are placed on me, the lower my ability to cope and the lower my energy level. So that reduces my motivation to exercise and also to cook at home versus, you know, just going out to eat.” *(SU30700)*   ***Lack of support for weight management in primary care***   - “[My primary care physician] usually tells me I should exercise more and I should lose some weight, and then I come back and nothing happens.” (*PA25965*) - “I don't know that I’ve gotten, that I feel like I’ve gotten much help from my primary care physician for any of these things, really, other than the depression […] Everything else is just the same old, ‘you need to reduce your calories, you need to eat this many vegetables a day, all the stuff I already know,’ so you kind of go, ‘yeah, yeah, yeah, fine.’” (*MV09434*) - “In my current healthcare, I basically go see my doctor, and I get shamed for being too heavy. She's just, like, ‘Oh, you should really lose weight.’” (*PA30976*) | **Control Group**  ***Lack of time***   - “I think it’s just my level of busyness. So, if I know I have a very busy day, you know, I think those are the days where I’m just, like, I don’t even have time to track the food. Or, I’ll do it later. And then, you know, I don’t get back to it. So, the busyness of my workday probably is the biggest impact or the deterrent to tracking my food.” (*MV00993*) - **“**The big challenge for me is I travel all the time for work. I don't find hotel gyms and stuff all that great, so when I'm traveling a lot, it's just very hard to do.” (*PA22969*)   ***Stress***   - “I have a person now living in my house where I didn’t before. That’s very taxing, to say the least […] Their mother died and father died in the last year. So, they’re a little edgy still. So, they’re still kind of trying to deal with it. So, you have to walk on pins and needles in your home, and it’s kind of difficult at times. You try to do the best you can. But it seems that I get madder because you can’t get away from it.” (*PA30091*)   ***Challenges with mood management***   - “I function, but not all that well. […] Not as well as I had hoped. […] Just general, my mood is still very low. I’m still very stressed. I worry about everything. I don’t seem to be able to focus on anything.” (*MV03729*) - “Since I have a lot of chronic pain issues, I can get depressed every now and then. For me, that just means I’m very low energy, and I don’t go out very much. I’m not very motivated to do really anything when I’m really low. When it’s moderate, I can do the basic things and see friends and things like that, but that’s about it. So, the past six months, I’ve been up and down.” (*PA22014*)   ***Challenges with adopting healthier behaviors***   - “Once I got into a rhythm, I could stick with it, but then if I start drawing exceptions, I very quickly fall off the wagon, and then I just kind of get into a pattern of not doing it anymore.” (*PA22969*) | **Control Group**  ***Lack of time***   - “There just never seems to be enough time to do everything I want. […] Usually my walk that I would like to do gets dropped. If I don't get it done first thing in the morning, then it just…yeah. So I would say classes I typically go to, but the stuff that's not scheduled, often just drops.” (*MV01109*) - “I have access to facilities and things. It’s mostly just been a time issue. I haven’t found a good way around that.” (*PA28460*)   ***Stress***   - “We just bought our second home, […] and it was very stressful trying to get it. So by the time we would leave the bank at 8 o'clock at night, we don't have time—we have the kids—to go home and make dinner, so, like, ‘let's just stop and get pizza. Let's stop and get Taco Bell. Let's stop and get Jack in the Box.’ So, those types of things really didn't help, and then, we have things in boxes. We can't make dinner now for so many weeks, so a lot of things have added up.” (*SU335500*)   ***Challenges with mood management***   - “We're trying to improve my mood, but I don't really know how to come to grips with that.” (*MV03729*)   ***Challenges with adopting healthier behaviors***   - “I’m a teacher, so if there was something I could go to right after work, I feel like I would participate more. If I go home, I’m less likely to be motivated to go back out to a meeting or go out to a class.” (*MV00993*) | **Control Group**  ***Lack of time***   - “I have a special needs child with a medical condition, and so I spend a lot of time taking care of him and taking him to all his various appointments. So, there’s not a lot of time left for me.” (*PA21658*) - “There was no way I was going to be able to see [a therapist] during the day, get away from work. It would have been a challenge to get there from home. Getting through traffic, getting there, getting back during the day was just out of the question.” (*MV03729*)   ***Stress***   - “It’s been a stormy time in my family with several illnesses, and my family lives in Florida right now […] so the hurricane result of that has been pretty stressful. […] It’s just there’s been a lot of it.” (*MV05164*)   ***Challenges with mood management***   - “[I’m] not very satisfied because instead of losing weight, I think I gained a lot more. But I think it had a lot to do with my depression and my mood because last year around this time, that's when, yeah, at the end of May, that's when I lost my job, and so my depression plummeted. Yeah, my mood plummeted, and so, yeah, from then on things went downhill.” (*PA22725*)   ***Challenges with adopting healthier behaviors***   - “I have not been focused and disciplined about how I eat or about exercising regularly.” (*MV03729*) |
