# Supplementary material for: Using RE-AIM to examine the potential public health impact of an integrated collaborative care intervention for weight and depression management in primary care: Results from the RAINBOW trial
Source: PLoS One. 2021 Mar 11;16(3):e0248339. doi: 10.1371/journal.pone.0248339 (PMC7951877; doi:10.1371/journal.pone.0248339)
Supplement: S3 Table — aEach quote is identified by the stakeholder type, stakeholder ID (if available), and timepoint. Condition assignment (intervention or control) is specified for participants at 6, 12, and 24 months, but not at baseline (pre-randomization). For participants, baseline refers to pre-randomization at enrollment, 6m refers to the end of the intensive treatment phase (6 months after enrollment); 12m refers to the end of the maintenance phase (12 months after enrollment); 24m refers to the end of the treatment follow-up phase (24 months after enrollment). For other stakeholders, baseline refers to the beginning of trial; 12m refers to 12 months after trial start; 24m refers to the end of the trial. (DOCX) [file pone.0248339.s004.docx]

**S3 Table. Supporting Quotes for Themes Identified for the Adoption Dimension^a^**

| **Theme** | **Baseline** | **12m** | **24m** |
| --- | --- | --- | --- |
| - 1. **Return on investment and cost-effectiveness** | **Intervention Staff**   - “I think if the organization can see the rationale, see how much money that it's actually saved, then maybe they would hire someone part time as a consulting psychiatrist, but they have to see the budget. They have to see the benefit of that in the budget.” (*I03)*   **Clinical Staff**   - - - “Leadership will look at it and say, ‘okay, how this program is going?’ I know that, okay, we have good results, it’s effective, and all these things, but they also look carefully at the financial part of it and how it’s going to cover the cost-effectiveness […] especially something that insurance is not going to cover.” *(A01)*     - “The biggest issue is about the scalability of putting this operating model in clinical practice with an eye that there is a return on investment.” *(A03)* | **Intervention Staff**   - - - “Part of the whole cost is the challenges with scheduling people, having them show up. People that cancel appointments have to be rescheduled, that drop out and are unresponsive. And all of that back-room work of trying to get participants in to work with them that the coach has to do that is time consuming and labor intensive. […] Maybe it becomes more cost-effective [when] you're only working with participants that have a strong motivation and desire and the ability to make appointments, keep up appointments, and stay with the process.” (*I01)*     - “I think people would want it. I think the question is, how you can operationalize it and pay for it, I think is the major thing.” (*I02)* | **Intervention Staff**   - “In terms of getting payers to reimburse, how are they going to reimburse the health care coach, the psychiatrist, all the different members involved, and work all that out, actually?” *(I03)*   **Clinical Staff**   - “It could be that we shift it a little into more of a primary care model based on some findings from the RAINBOW study. […] That's something that we're trying to do […] show them the raw data and show that this is quite workable and something that could spread.” *(PCP02)* |
| - 1. **Physician buy-in** | **Intervention Staff**   - “There would have to be a system for referring patients, there would have to be, I guess. That's a challenge. Physicians in primary care and physicians in behavioral health, they would be the ones that would be referring patients to this. So the first challenge would be having buy-in from them and having them see this as a valuable resource that they would refer patients to.” (*I01)*   **Clinical Staff**   - “It depends on […] how much hassle physicians might perceive it to be if the communication across the team is time-consuming. For some there’ll be, I think, at least initially, a concern about loss of their sense of being autonomous or in charge. […] If we believe that addressing mood and weight are the common path to addressing many of the other issues that are also on the table, then we have to figure out a way to frame this. Again, I think if there’s evidence that this works, I think it will be easier. […] If [a provider] thought if this really will work, then that actually makes the rest of her job easier, so it’s about proving the efficacy, confirming that there’s a sensitive, respectful way of approaching patients, that you’re not undermining the physician.” (*A02)* | **Intervention Staff**   - - - “I think we've had some times where the PCPs don't want to take our advice and things like that, and I think some of that is probably just the trust issue, because they don't know us. They don't really know who we are and so forth.” *(I02)*   - “I feel like the PCPs have been pretty open to recommendations, and I do think that it helped that I worked here before, and I had that relationship with some of the PCPs already. It might be different if I was an outsider coming in.” (*I03)* | **Intervention Staff**   - - - “The primary care has to be willing to trust the health coach and to allocate some things and trust the psychiatrist. […] If it were a new person coming in, if I were going to a clinic [where] I didn’t know the primary care doctors, I would want to meet with them, like weekly team meetings face-to-face, in the beginning at least, just to establish a rapport and a connection and trust.” *(I03)*   **Clinical Staff**   - “I think anything that requires more time on the physician’s side will be difficult to implement. I think teaching docs to do new things is difficult, too.” *(MD)* - “You’d have to come and talk to the doctors about it and see if they want to be involved in it, or if you want it to be implemented in primary care at a bigger level, I would say even discussing this at a department meeting—how everybody feels about it and at a bigger level than just one clinic level.” *(MD)* |
| - 1. **Very particular set of skills** | **Intervention Staff**   - - - “Finding qualified people, I think, is a challenge because, you need somebody that […] has a good understanding of nutrition and fitness but also is able to do the I-CARE problem solving, which is a specific set of skills, and finding people that are able to do that can be challenging, perhaps.” (*I01)* | **Intervention Staff**   - - - “I think the health coach actually has to have a very solid background in connecting with patients, having some level of counseling, plus having some global experience with dealing with depressed patients, anxious patients, and just their knowledge of health and nutrition, so I think attracting people that qualify can be difficult.” (*I02)* | **Intervention Staff**   - “Having an interventionist just dedicated to this role, it takes a lot of time. […] I see this position as there’s so much going on, that it’s almost a full-time position.” *(I04)*   **Clinical Staff**   - - - “It's a different training than they have for a lot of our staff right now, and somebody that has experience [as a] health coach doing this, I think that's more likely to have success. I'm trying to envision in the clinic who would do this and, in this format, the way you have it, I'm having trouble figuring out who would do this.” (*MD02)* |
| - 1. **Operational resources** | **Intervention Staff**   - - - “I think the manpower's here, in terms of primary care, and psychiatry. It's just a different way to coordinate things, I think.” *(I03)*   **Clinical Staff**   - - - “I think any kind of program like RAINBOW, if you’re going to implement it in the clinical setting, and especially at PAMF, you need to work very closely with the operation part. It’s not only working with a group of doctors or clinicians and nutritionists and all this. You need to work very closely with the operation in terms of finding the space and all these things.” (*A01)* | **Intervention Staff**   - - - “I think the team needs to be well-formed before and actually approach the primary care doctor and do a lot of education and [do] a lot of the organizational part, like organizing the workflow for the physician because I don't think PCPs are able to have the time to start things like that. […] I think there would be some sort of case manager within the primary care setting that could actually do outreach with patients and act as a liaison between the PCP and the team, and then the I-CARE team, obviously, but I do think there needs to be some support within the primary care setting.” (*I03)* | **Intervention Staff**   - - - “We were always scrambling to find rooms […] when our usual place wasn’t available. […] Not having a consistent room or a consistent place for a participant to go to, it affects them because they’re like, ‘oh, well, where am I supposed to go?’ and sometimes they get frustrated.” *(I04)* |
| - 1. **Helps meet demand for psychiatric and preventative care in primary practice** | **Intervention Staff**   - “I would think, if it's cost effective, and there would be logistical ways of doing it, that would not be disruptive. I don't see why they wouldn't want to do it.” (*I01)*   **Clinical Staff**   - - - “I think there would be a very high interest because this is an area […] that we're completely lacking, in terms of both weight management and mood disorder, so I think that it would be very welcome, especially if you can show that it has helped patients in the end result.” *(MD06)* | **Intervention Staff**   - - - “Most primary care doctors I deal with, I work with, would appreciate any form of help with mental health, because I think they're just overwhelmed and overworked, so I think most primary care doctors would be open to it.” *(I03)* | **Intervention Staff**   - - - “I think most places with primary care are going to be interested in this because they see so much of this, and I feel like there’s such a demand for psychiatric care […] especially in rural areas, so it’s hard for me to imagine any primary care clinic not being interested.” *(I03)*   **Clinical Staff**   - - - “All of our doctors are over-paneled, and I think we’re all a little bit short on time, right? For weight management and depression. So I think it would be helpful.” *(A)* |
