# Supplementary material for: Using RE-AIM to examine the potential public health impact of an integrated collaborative care intervention for weight and depression management in primary care: Results from the RAINBOW trial
Source: PLoS One. 2021 Mar 11;16(3):e0248339. doi: 10.1371/journal.pone.0248339 (PMC7951877; doi:10.1371/journal.pone.0248339)
Supplement: S4 Table — aEach quote is identified by the stakeholder type, stakeholder ID (if available), and timepoint. Condition assignment (intervention or control) is specified for participants at 6, 12, and 24 months, but not at baseline (pre-randomization). For participants, baseline refers to pre-randomization at enrollment, 6m refers to the end of the intensive treatment phase (6 months after enrollment); 12m refers to the end of the maintenance phase (12 months after enrollment); 24m refers to the end of the treatment follow-up phase (24 months after enrollment). For other stakeholders, baseline refers to the beginning of trial; 12m refers to 12 months after trial start; 24m refers to the end of the trial.* Note: PCP = Primary Care Provider. (DOCX) [file pone.0248339.s005.docx]

**S4 Table. Supporting Quotes for Themes Identified for the Implementation Dimension^a^**

| **Theme** | **6m** | **12m** | **24m** |
| --- | --- | --- | --- |
| - 1. **Engagement with and use of I-CARE components** | **Intervention Group**   - “There's the binder that we get that has the exercises in it. There are the videos that I started watching maybe two months into the program. I was visiting with the health coach […] and working on goals and trying to come up with ways that I could complete those goals. […] I’m doing the Fit Bit where my activity is tracked. I have been doing that before I started doing the study, so that was an easy thing to just keep doing, and then one of the exercises I was doing, one of the activities I guess you could say, was tracking my food, and I did that for probably about five months. But the last month I have not been doing that, and that's my own letting myself not do it, and the visits have gone away because that's how the study is going, right? So first you see them very frequently, and then you see them less frequently and less frequently, and then it gets to be on the phone as opposed to being in person." *(MV09434)* | **Intervention Group**   - “I think the once-a-week kept me more accountable. […] There would be something in the back of my mind, like, ‘shit, I've got to see him.’” *(PA21735)* | **Intervention Group**   - “I was seeing a personal trainer. I started earlier this year, and I did that for about four months until I got really busy at work, and then I stopped going, so I haven't picked it back up yet.” *(MV07554)* - “It was just up to me to follow through with what was given to me, and I didn’t pick up responsibility or prioritize it enough for myself.” *(SU33352)* |
| - 1. **Coordination of medication management** | **Intervention Staff**   - “We had made a suggestion for an anti-depressant medication […] but the PCP* didn't agree with that. […] They didn't agree with the recommendation. And, then eventually they did order something a little bit different. So, that not agreeing with the recommendations we had made. Or, not knowing. There's been an instance or two where they didn't know where to find the recommendation. So, a participant made an appointment with them, and went to see the PCP. But, they didn't know what to do then, because they didn't know where to find the information in the medical record, where we had written the recommendation notes.” (*I01*) - “[Coordination related to medication management] takes a couple of weeks. By the time the patient gets in the system, we see them, [the consulting psychiatrist] makes a recommendation, it goes back to the patient. Then the patient goes and sees the PCP, and maybe there's another note. So, it takes a few weeks, whereas, if the patient saw me in the office, and I wasn't sure about something, I could send a note to [the consulting psychiatrist] at the first visit and have an answer in three days, theoretically.” (*I02*) - “One thing that's made [balancing my regular workflow with I-CARE] better, too, is with the documentation. We came up with a note template that we send to the PCPs with our recommendations, and I do that actually during the [case review] meeting. So, we just started that, and that's really helpful, because if I leave the meeting, it just doesn't get done. I mean, it's fresh in my mind, and I just write the note as we're talking about it. That's been helpful.” (*I03*) | **Intervention Staff**   - “I think that the inter-team staff messaging has worked fine. No drawbacks to that. And I think very early on in the program there was some pushback from physicians, PCPs who thought they were getting too many staff messages around this, which is why we kind of changed our policy a little bit. But I think recently we haven’t heard anything. No drawbacks.” *(I01)* - “I think we've had some times where the PCPs don't want to take our advice and things like that. And I think some of that is probably just the trust issue, because they don't know us. They don't really know who we are and so forth.” (*I02*) - “I think [coordination related to medication management has been working] pretty good. I think sometimes there is easy dose adjustments that are made. I think it often takes more time than it needs, because we kind of check with the patient and they come back to us. And then the primary [care provider] may streamline that a bit, so that's gotten a little bit better.” (*I02*) | **Intervention Staff**   - “It’s been working out pretty well. I—what tends to happen is if there’s any questions about medication, then [the consulting psychiatrist] takes a look at the chart, she makes a recommendation, I go back to the participant and I talk to them next time, and then at the next meeting and let them know about our recommendation. More times than not, the patient will go back and talk to their doctor and let them know that the psychiatrist has made this recommendation.” (*I04*) - “[The health coach] did a really good job of summarizing and going through the information that was important, and there was kind of a learning curve with that, but I think we got better and better at that as the study went on, and it was really—I felt like the meetings were very efficient and informative.” (*I03*) |
