# Supplementary material for: Using RE-AIM to examine the potential public health impact of an integrated collaborative care intervention for weight and depression management in primary care: Results from the RAINBOW trial
Source: PLoS One. 2021 Mar 11;16(3):e0248339. doi: 10.1371/journal.pone.0248339 (PMC7951877; doi:10.1371/journal.pone.0248339)
Supplement: S5 Table — aEach quote is identified by the stakeholder type, stakeholder ID (if available), and timepoint. Condition assignment (intervention or control) is specified for participants at 6, 12, and 24 months, but not at baseline (pre-randomization). For participants, baseline refers to pre-randomization at enrollment, 6m refers to the end of the intensive treatment phase (6 months after enrollment); 12m refers to the end of the maintenance phase (12 months after enrollment); 24m refers to the end of the treatment follow-up phase (24 months after enrollment). For other stakeholders, baseline refers to the beginning of trial; 12m refers to 12 months after trial start; 24m refers to the end of the trial. (DOCX) [file pone.0248339.s006.docx]

**S5 Table. Supporting Quotes for Themes Identified for the Maintenance Dimension^a^**

| **Theme** | **6m** | **12m** | **24m** |
| --- | --- | --- | --- |
| - 1. **Intent to continue strategies to manage weight and mood** | **Intervention Group**   - “I'm very likely to continue doing the things that the program has suggested to the degree that I can manage and do it, the caveat being that I had already being doing most of them anyway, so it's not a drastic change.” *(PA21152)* - “I'd say I’d definitely continue the problem-solving exercises that I’ve gone through with the health coach, coming up with several things you can do to accomplish your goal and then deciding what would be the most efficacious. […] I’d say that's the most single important thing that I’ve learned.” *(MV09434)*   **Control Group**   - - - “I’m not really doing much of anything, so that’s the thing, just walking, but not far enough, so it’s just nothing’s really working out.” *(PA30091)*     - “I think at some point I’d like to be able to believe that I would be able to. I’m definitely willing. Can? Yes, I would say so. But I think I have to figure out strategies that keep things long-term.” (*PA22014*) | **Intervention Group**   - “[I’m] very, very likely to continue it because I think the program is pretty cool. I enjoyed it. […] I think it would be very easy for me to continue with this. […] I think the habits of knowing the information that has been provided and knowing what I have to do make a lot of sense. I just have to keep charging forward on it.” *(MV03987)* - “I find that if I try to relax myself before I go to bed, it’s easier for me to fall asleep, but it’s still something I have to try to continue with as a daily regime for myself and the hope is that I can continue it.” (*SU33352*)   **Control Group**   - *“*I’m fairly willing to do anything to address my weight. To address my mood, I don’t know. My own personal happiness and those kinds of things are not super important to me.” *(PA29689)* - *“*I would like to lose 15 to 20 pounds. […] Not sure if I will just be working on that at home or go back to Weight Watchers. I haven’t quite decided about that.” (*MV00993*) | **Intervention Group**   - “I’m pretty likely to keep trying. My mood has definitely improved. […] I think I’m continuing to do some of those, definitely the exercise and the journaling about how much I exercise.” *(PA24005)*   **Control Group**   - “I have debated seeing a psychiatrist to try medication again. I used to take Celexa, but I didn’t feel like it was working that much, so I went off of it last fall. I have mixed feelings about trying it again.” (*PA21658*)   **Intervention Staff**   - “Even when we start with the lifestyle changes, the problem solving is always the most consistent thing because we’re always problem solving around different things, so that’s what stays with them.” (*I04*) |
| - 1. **Level of confidence in ability to continue strategies to manage weight and mood** | **Intervention Group**   - “It’s up to me. I have the tools, and I’ve been given some good tools and some good goals, and now I just have to do my part, you know?” (*MV01332*) - “[Food] is my drug of choice and using things like high-fat sugar things, ice cream […] I could go out of here and find myself saying ‘oh, God, I've got to have ice cream,’ but I don't expect that to happen. […] It is better than it was, and so that’s been good because that's a big issue.” (*PA26904*) - “I think the harder [part] for me was to take the time, make time to concentrate on exercising. The easier part was actually realizing to eat better, and it brought my awakening to changing, having the options of the good and the bad.” (*MV10489*) - “I think I myself am failing your program, and I know they're disappointed that I'm not reaching that seven percent.” (*PA23283*)   **Control Group**   - - “I have a tendency to think all or nothing and that kind of dooms me.” (*PA29182*) | **Intervention Group**   - - “I think I’m pretty confident, yeah. […] I’ve learned a lot of what to do and what not to do, so it’s just a matter of the control and just if you fall, get up again.” (*MV06639*)   - “Although I’m off track right now, I know that I can go back and do the things that I did prior because it did work with the follow-through and the support that I had during the program.” (*SU33352*)   **Control Group**   - “I would like to be able to exercise, but that does not really seem to be in the cards right now. […] I just eat a lot. Anyway, I don't know. I don't know what can be done that my body isn't preventing me from doing.” *(MV05164)* | **Intervention Group**   - “I wrote down what I wanted to lose originally, and I wasn’t near that, […] so, I’m like, ‘come on, man, you can do this.’ […] For me, it’s keeping up the activity and building it into my day, because then even if I slip a little bit on the food—but again, it’s just a constant balance. *(Interviewer: How confident do you think you are that you can keep doing those things?)* I say, a nine.” *(PA21735)*   **Control Group**   - “The weight is just like a battle forever. […] [I have] multiple problems, like four or five, trying to deal with them all at once, and I don’t really know which one to deal with completely because none of them you can really finish. […] So I stress myself out.” *(PA30091)* |
| - 1. **Maintenance strategies** | **Intervention Group**   - - - “I’m still committed to trying to do something about the calorie intake. I really want to do a week or two of careful measurement of the calories I’m taking and then try and actually make some changes to the diet to help that.” *(PA27047)*     - “I think I need to plan time on my calendar of when I’m going to get activity and exercise, or it won’t happen. I’m hoping to go for a hike every weekend with my family, and as far as diet choices, […] I will try to just get water instead of other drinks when I eat out and try to plan ahead, so I don’t end up having to get fast food because of time constraints.” *(SU30700)*   **Control group**   - - - “I can’t think of anything for those, any long-term strategies for improving, other than just doing it, which is the challenge.” *(MV03729)*     - “I'm trying to figure out what days I can go to the gym consistently and do exercise also at home, so I'm trying to reconfigure my whole routine.” (*PA21658*) - “I try to eat the right stuff. I think that I'd be able, like I said, I attempt to do exercise. It doesn't always work, and the only exercise I can do consistently at all is the walking thing. […] I would definitely seek professional help if I needed it again. […] But as far as the weight, I haven’t really addressed it at all.” (*MV05164*) | **Intervention Group**   - - - “I really need to plan snacking. I just need to plan. I plan my breakfast, lunch, and dinner, and it's the in-between that's unplanned, so staying with the exercise and the planned eating, don't eat after 8:00, then don't beat myself up.” *(PA21735)*     - “I think breaking down the exercise into doable pieces, instead of trying to find an hour, to find five minutes to do one of the exercises, to piece those in during the day. That's a really good strategy. I just think breaking it down into doable parts has been the primary thing for me.” *(PA2328)*   **Control Group**   - - - “I’m not sure what I’m going to do about that, but I guess maybe go to the gym that I belong to, and like I say, I’m going to change my diet, and I have a little bit, but I need to focus more on that and not eat the wrong things. Have one piece of pizza instead of four.” *(PA27432)*     - “My hope is that this summer when work is different, I will be able to find time to get in more regular workouts without the trainer, but right now I can only squeeze workouts in when I’m with him, and other than that, it’s almost impossible.” (*PA29689*) | **Intervention Group**   - “I’m scared to death because I want to maintain. I want to lose a few more pounds, but I want to keep doing what I’ve been doing, so I’m just going to be holding on to those two things for dear life, the app and the Fitbit, and then I’m going to keep signing up for classes. That gives you your exercise built in. I mean, I still try and do stuff on the weekend, but that’s built-in, and I signed up for the Be Well thing, and I had my—I told you, they measured me this morning, and I got my blood drawn. So now that everything’s much more in control, I want to focus on raising my HDL cholesterol. That’s the only thing that I can tweak, I think. So, I’m going to—I just read articles. I eat all the food you’re supposed to already for it, so I don’t know what I’m going to do, but anyway, that’s my goal.” *(PA21735)*   **Control Group**   - “I've just always found that, for me, motivation is something I know I have to do and just keep physically active and just trying keeping that going all the time.” (*MV0993*)   **Intervention staff**   - “I find that a lot of participants will start scheduling in things a little bit more that they find important to them whether it be eating habits like planning their meals or packing up their meals or whether it be exercise, if they find it important, the more likely they’ll plan it in. So, having them come up with that plan helps them kind of continue with the content that we covered in the program.” (*I04*) |

| **Theme** | **24m** |
| --- | --- |
| - 1. **Better mental health care needed for maintenance** | **Intervention Group**   - “I would rather my primary care physician be a coach […] I mean, because often I’m not sick. […] I rarely need to go to a doctor. There’s nothing wrong with me. My insurance is paying for all these people to be available, but they’re not available to do the things that I need them to do, but, so, if behavioral health people were treated in the same way as my primary physician or my allergist, that would be different. That could be more useful to me. Having a resource to deal with my ongoing depressive issues, but at the same time, someone who could lead me along to the next step. Seeing where I’m at and showing me what I need to do.” (*MV04106*) - “They could employ psychologists instead of just referring you out or letting you see people who kind of just want to check you off your list, that would help. And make it easier to see a psychiatrist. It takes a long time. And medical doctors also. General practitioners are afraid to talk about depression. They just think you’re going to kill yourself, and once you tell them you’re not, then they’re, like, ‘okay, well, here’s a pill,” and they’re too afraid to even sit down and talk for a while, five minutes about how things are difficult.” (*MV04933*) - “I think finding a psychologist is super difficult. […] I know that's not PAMF's fault, but I also know that it takes weeks and weeks to get more names, and it's just that whole process is complicated, and if PAMF could help me navigate that a little better, that would be really cool.” (*MV07554*)   **Clinical Staff**   - “Has it been discussed to do a group appointment or shared medical appointment with patients, to bond with each other or this experience where they have a little support group? […]. I would think that they have their little club or little ongoing—that possibly they could benefit from each other?” (*MD01*) |
| - 1. **Ongoing staffing resources needed to maintain program** | **Intervention Staff**   - “I think one [barrier] is just everyone in primary care clinic pretty much feeling like stretched to maximum capacity already, just getting people to, getting other staff, like getting someone to facilitate the support group, for example.” (*I03*)   **Clinical Staff**   - “We need the resources to provide to patients and that it's potentially quite possible to do that within primary care. I think that just helps patients because it’s more integrated into their, sort of the “one doctor” thing. […] Having seen having a coordinated resource that works together with the primary care doc, I think that’s an ideal position.” (*PCP02*) - “The advantage of this is that you’re actually having one person coaching the patients for two conditions. I think naturally it’s advantageous. It just seems more efficient.” (*A03*) - I think the biggest thing is that health coach, have staffing in that position, educating them, making sure they are a quality health coach and then making sure that they are happy in their role, and if they were to stay in that role, then that could potentially be a long-term thing […] because we have so many staff who are leaving and how the high staff turnover—and so that personnel need. (*MD01*) - “It’s the beauty of the staff we have is that […] they connect with the patient, and they have different skills than the physician, and so patients will tell them things that they might not tell us and vice versa. It can really help the patient. […] So, I really think that diversity is important on a team.” (*MD*) |
| - 1. **Health system role in supporting patients** | **Clinical Staff**   - “I think the health system’s role is really one of promoting patients’ health management in this phase […] to monitor their progress and then adjust treatment intensification accordingly, and then, just be mindful connecting patients to the resources that they need for optimal outcomes.” (*A03*) - “It would be probably be helpful to have some smart phrases to help follow up, because if we were going to adopt it, I think the primary care physicians would do some of the follow up, and so it’d be nice to have some smart phrases with specific questions to ask the follow up for these patients.” (*MD*) |
